# Genome sequence analysis of an extensively drug-resistant *Acinetobacter baumannii* indigo-pigmented strain depicts evidence of increase genome plasticity

**DOI:** 10.1038/s41598-018-35377-5

**Published:** 2018-11-16

**Authors:** German Traglia, Kevin Chiem, Brettni Quinn, Jennifer S. Fernandez, Sabrina Montaña, Marisa Almuzara, María Alejandra Mussi, Marcelo E. Tolmasky, Andres Iriarte, Daniela Centrón, María Soledad Ramírez

**Affiliations:** 1 Instituto de Microbiología y Parasitología Médica (IMPaM, UBA-CONICET), Facultad de Medicina, Universidad de Buenos Aires, Buenos Aires, Argentina; 2 Center for Applied Biotechnology Studies, Department of Biological Science, California State University Fullerton, Fullerton, CA USA; 3 Laboratorio de Bacteriología, Hospital Interzonal de Agudos Eva Perón, San Martín Buenos Aires, Argentina; 4 Centro de Estudios Fotosintéticos y Bioquímicos (CEFOBI- CONICET), Rosario, Argentina; 5 Laboratorio de Biología Computacional, Dpto. de Desarrollo Biotecnológico, Instituto de Higiene, Facultad de Medicina, Universidad de la República, Montevideo, Uruguay

## Abstract

*Acinetobacter baumannii* is a multidrug resistant nosocomial pathogen that shows an outstanding ability to undergo genetic exchange, thereby acquiring different traits that contribute to its success. In this work, we identified genetic features of an indigo-pigmented *A*. *baumannii* strain (Ab33405) that belongs to the clonal complex CC113^B^/CC79^P^. Ab33405 possesses a high number of genes coding for antibiotic resistance and virulence factors that may contribute to its survival, not only in the human host, but also in the hospital environment. Thirteen genes conferring resistance to different antibiotic families (trimethoprim, florfenicol, β-lactams, aminoglycosides and sulfonamide) as well as the *adeIJK* genes and the capsule locus (KL) and outer core locus (OCL) were identified. Ab33405 includes 250 unique genes and a significant number of elements associated with Horizontal Gene Transfer, such as insertion sequences and transposons, genomic islands and prophage sequences. Also, the indigo-pigmented uncommon phenotype that could be associated with the monooxygenase or dioxygenase enzyme coded for by the *iacA* gene within the *iac* cluster was probably conferred by insertion of a 18-kb DNA fragment into the *iacG* gene belonging to this cluster. The Ab33405 genome includes all type VI secretion system genes and killing assays showed the ability of Ab33045 to kill *Escherichia coli*. In addition, Ab33405 can modulate susceptibility antibiotics when exposed to blue light.

## Introduction

*Acinetobacter baumannii* is an important nosocomial pathogen that causes a variety of diseases, such as pneumonia, skin and soft tissue infections, meningitis and bacteremia among others^1–8^. Its ability to form biofilms facilitates its survival for long periods on inanimate surfaces^7–9^. *A*. *baumannii* strains are usually multidrug drug resistant, which makes it a hard-to-treat pathogen that causes high morbidity and mortality^7,10–14^.

In 2013, we reported an outbreak of hospital infection caused by an indigo-pigmented *A*. *baumannii* strain that began in the traumatology service ward of an acute care institution in Argentina^14^. To our knowledge, this was the only report of an indigo-pigmented *A*. *baumannii* strains outbreak. Although the nature of this pigment remains to be elucidated, it could be related to the activity of a monooxygenase or dioxygenase enzyme encoded by *iacA*^15^. The strain was multidrug-resistant, belonged to the CC113^B^/CC79^P^ and harbored the transposon Tn2006, a class 2 integron, AbaR-type islands, IS125, IS26, *strA*, *strB*, *florR* and the small recombinase IS*CR2* associated to the *sul2* gene proceeded by IS*Aba1*^14^.

Ninety-five complete *A*. *baumannii* genome sequences and more than 2,000 draft genomes are available in the GenBank database. Most of the genome studies were done with strains belonging to the international clonal lineage (ICL) 1 and 2^16–18^. In those studies, genomic comparison between different strains and/or different lineages, particular features of the studied strains, and the capability of this bacterium to develop resistance to antibiotics were explored^17^. Snitkin *et al*. showed that strains belonging to ICL2 have an elevated number of SNPs located in gene clusters related to immune evasion^19^. In addition, a complete characterization at the genomic level of a hypervirulent strain (LAC-4), that does not belong to the ICL1 or ICL2, was described. This study showed particular genetic features and the high genome plasticity of this strain^20^. Comparative genomic studies of *A*. *baumannii* exposed the genetic variability across all *A*. *baumannii* strains^16,21,22^. Collectively, genome studies can contribute to gain further knowledge in the pathogenicity, physiology and molecular basis of antibiotic resistance and spread of resistance determinants in this pathogen.

Therefore, the aim of this study was to identify the genetic features of an indigo-pigmented *A*. *baumannii* strain (Ab33405) that belongs to the CC113^B^/CC79^P^.

## Results and Discussion

### Whole genome comparison

The assembly of the Ab33405 strain yielded 3,923,578 bp with a G + C content of 39.06% and 3749 predicted protein-coding sequences with an average gene length of 900 bp (Table 1). The number of predicted proteins, as well as the average length of the genes, is comparable with other previously sequenced genomes (Table 1). The Ab33405 strain belongs to the *A*. *baumannii* species, as stated by the phylogenetic analyses and the ANI score (e.g. 98.24± 1.80% with ACICU and 98.14 ± 1.97% with AYE strain).

**Table 1:** General features of Ab33405 compared with reference genomes.

Strain	Ab33405	ATCC 17978	AYE	ACICU
Size (Base Pairs)	3892826	3976747	3936291	3904116
Plasmids	ND	2	4	2
G + C contents	39.07	38.94	39.40	39.03
Protein-coding sequences (CDSs)	3741	3787	3607	3670
Insertion sequences	7	14	33	14
Average gene length	900	888	951	929
rRNA operons	6	5	6	6
tRNA	62	69	72	64

When we compared Ab33405 genome with seventeen reference genomes in the GenBank database (see Supplementary Table S1), 1,886 conserved gene families and 250 unique genes were identified in our strain. Among the unique genes, we found many phage related genes and coding sequences of unknown function.

**Figure 1:**
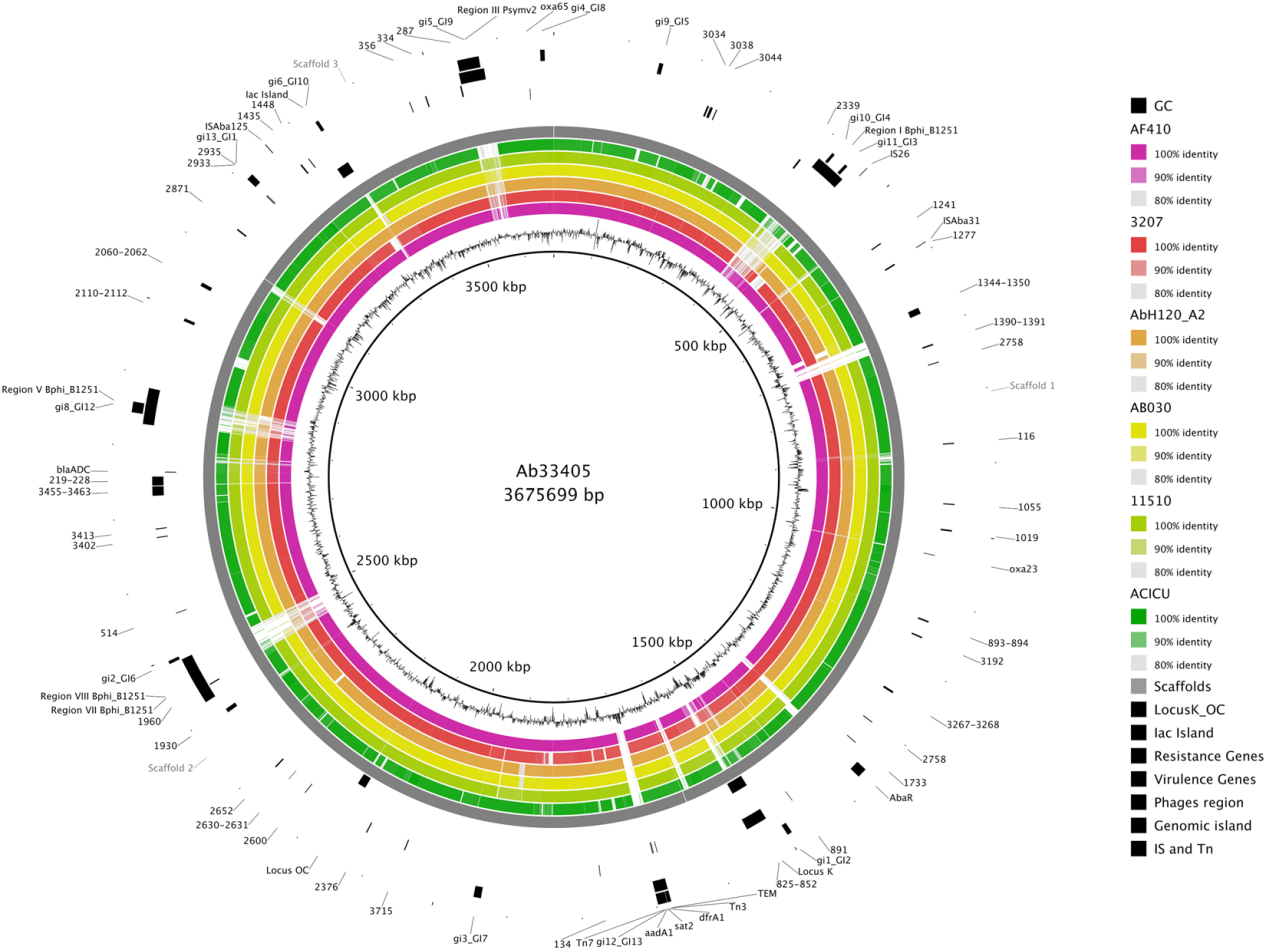
Comparative genomics circular visualization of Ab33405 with selected closely related strains plus the genome of ACICU strain. Ab33405 assembly was ordered using RAGOUT software in three scaffolds (Outer grey circle) and used as reference in BRIGS. The inner circle shows the GC content of the reference sequence. Blast comparisons with strains are shown ordered according to the observed phylogenetic distance, from inside (close) to the outside rings: AF401, 3207, AbH120_A2, AB030, 11510, and ACICU. The position of locus OC and K, the Iac island, resistance related genes (*bla*_OXA-23_, *bla*_TEM_, *dfrA1*, *sat2*, *aadA1*, *bla*_ADC_, *bla*_OXA-65_), virulence genes (numbers codes described in Supplementary Table S2), phage regions, genomic islands and mobile elements are indicated. Note that not all genes described in the text and identified among the assembled contigs could be mapped into the three main scaffolds presented in this figure. The total scaffolding in this figure comprise 3,675,699 base pairs.

Interestingly, these 250 singletons include many putative virulence genes (Fig. 1 and see Supplementary Table S2), such as *vipA* and *vipB*, which are a part of the type VI secretion system (T6SS). Also, we identified two copies of the gene that codifies for the HecA adhesin protein. This protein is known to contribute to the attachment, aggregation, epidermal cell killing and virulence phenotypes in *Erwinia chrysanthemi*^23^. Other unique, relevant genes are those related to antibiotic resistance, such as *bla*_TEM-1_, and elements related to horizontal gene transfer (HGT), such as prophage sequences. Many of the antibiotic resistance genes were absent in very closely related genomes. The genes *aadA1*, *sat2*, *dfrA1* and *bla*_TEM-1-like_ formed a gene cluster recently acquired, explaining the G + C content profile of the region (Fig. 1). Moreover, 22 hypothetical proteins with unknown functions were also found. Most of the unique hypothetical proteins identified were flanked by prophage sequences. Analyzing the hypothetical proteins, we observed that two possessed the domains of a wall cell-associated hydrolases, which was recognized to be involved in cellular invasion^24–26^.

A wider comparative genome analysis was done within the *Acinetobacter* genus. All draft and complete genomes of the genus *Acinetobacter* were downloaded from GenBank (available August, 2017). This original database comprised 2,545 genomes of the genus (see Supplementary Table S3). Among these, 95 genomes were sampled based on the sequence identity of highly conserved protein-coding genes (see Supplementary Table S4). The final dataset comprises 95 genomes, assumed as representatives of the genetic diversity of the genus, plus Ab33405. A comparative genome analysis was done and the pan-, soft core- and core-genome were estimated (see Supplementary Fig. S1). The estimated core genome of the genus consists of 772 homologous gene families, while the soft-core genome, which includes all clusters shared by 95% or more of the genomes analyzed, comprises 1,396 homologous gene families. According to the comparative analysis and considering the monophyletic distribution of genomes in the phylogeny shown in Supplementary Fig. S2, a preliminary estimation of the conserved set of homologous families in *A*. *baumannii* species is 2,150.

### The phylogenetic position of Ab33405

Putative orthologous genes among the 96 sampled genomes were identified and used for defining the phylogenetic position of Ab33405 strain in the genus (see Supplementary Fig. S3). As expected, the Ab33405 strain clustered close to *A*. *baumannii* species strains (Red box in Supplementary Fig. S2) and within the *A*. *calcoaceticus-baumannii* complex (Green box in Supplementary Fig. S2), in both cases with statistically significant support (Node support >50%). We identified two *A*. *baumannii* strains (146457 and 348935) that cluster close to *A*. *gyllenbergii* and *A*. *lwoffii* species. The observed phylogenetic position of these genomes is relatively distant from other *A*. *baumannii* suggesting a miss identification of these genomes in the database.

Twenty-one genomes from the *A*. *calcoaceticus-baumannii* complex included in a robust monophyletic cluster were selected for further analysis (Green box in in Supplementary Fig. S2). These genomes were back tracked to the original sampling, and subsequently all closely related genomes were retrieved from the original database. The total number of genomes analyzed in the second phylogenetic analysis was 2,293, including: 2,055*A*. *baumannii*, 15*A*. *calcoaceticus*, 2*A*. *lactucae*, 33*A*. *nosocomialis*, 3*A*. *oleivorans*, 122*A*. *pitti*, 3*A*. *seifertii*, and 60 genomes of unspecified species. Almost all *A*. *baumannii* strains clustered as a monophyletic group with statistically significant support (see Supplementary Fig. S3), while the same is observed for the other species included in the analysis. Ab33405 strain clustered close with other strains of the ST 79, two strains of the ST 422, and ST 156 in the same monophyletic group. In both cases, there is difference in only one allele, *fusA* gene and the *rpoB* gene, respectively (see Supplementary Table. S5).

### Antibiotic resistance determinants and related mobile elements present in Ab33405 genome

The presence of antibiotic resistance genes in Ab33405 was observed in the central, also known as core (intrinsic genes), and in the accessory (acquired genes) genomes. It is well known that the acquisition of genetic determinants in *A*. *baumannii* is crucial to the evolution of this pathogen. A combination of different mechanisms -such as transformation, conjugation and transduction- and a variety of elements play a role in this process. Key elements directly involved in the acquisition of genetic material are ISs and transposons, genomic islands, integrons and plasmids^7,27^.

Genes involved in resistance to trimethoprim, florfenicol, β-lactams, aminoglycosides, and sulfonamides were found in the Ab33405 genome. The *bla*_ADC-like_ and *bla*_OXA-65_ *(bla*_OXA-51-like)_ genes, which are intrinsic genes in *A*. *baumannii*, were identified in the Ab33405 genome without ISs upstream or downstream. This genetic context has been associated with a basal expression of *bla*_ADC-like_ and *bla*_OXA-65_, which would only allow weak β-lactamic hydrolysis^28–30^.

**Figure 2:**
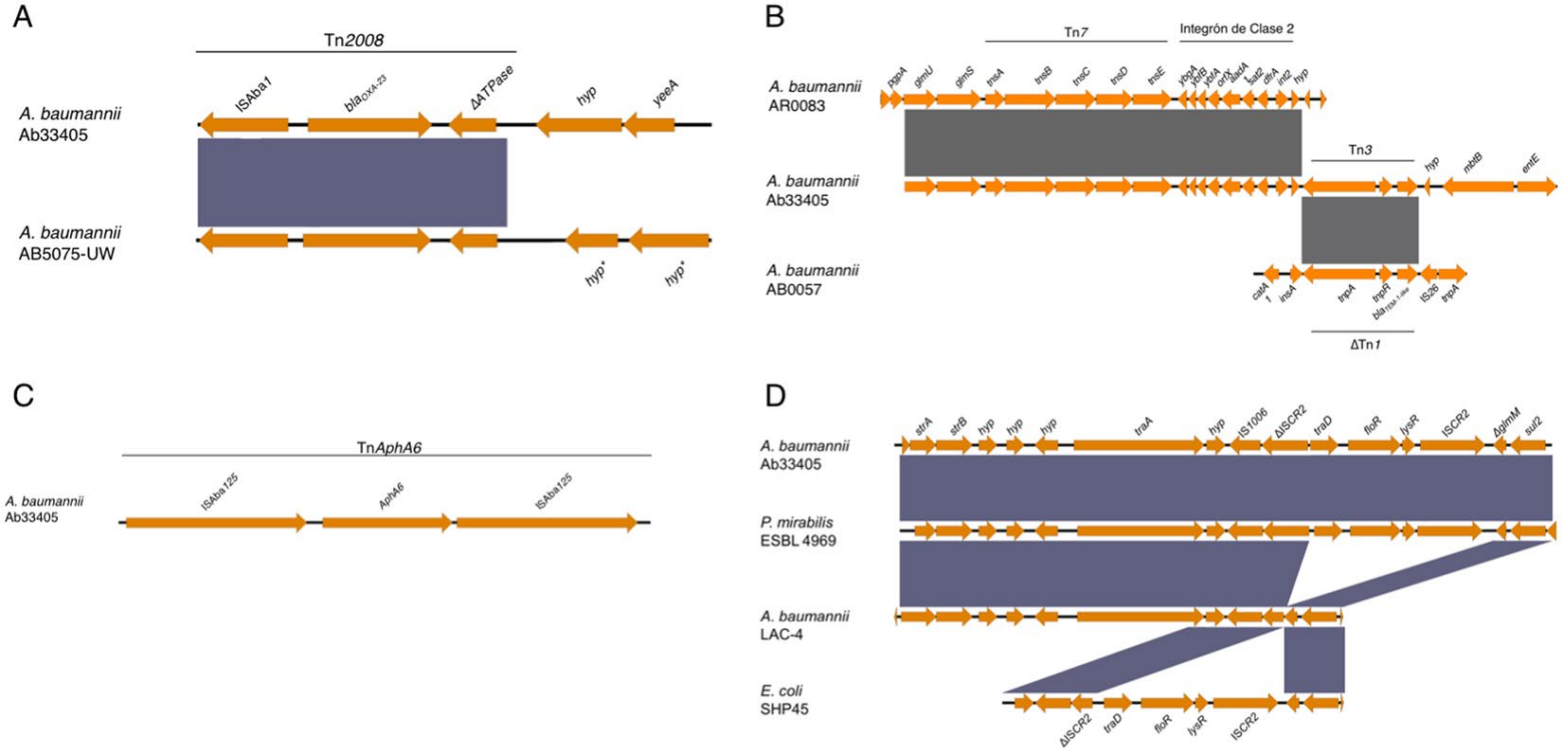
Schematic representation of genetic structures of different mobile elements associated with antibiotic resistance genes on *A*. *baumannii* Ab33405. The genetic structures were:(**A**) The Tn2008 transposon (**B**) In2:7 and Tn3 transposon, (**C**) Tn*AphaA6* transposon and (**D**) the genetic structure with ISCR2 and IS1006 insertion sequences and *floR*, *strA*, *strB* genes associated with antibiotic resistance phenotype. Every structures were compared to the most similar structures present in the GenBank databases. The (*) represented the presence of phage sequences. The graphic representation was made using the EasyFig. 2.2.2 software.

The remaining antibiotic resistance genes identified in the genome of Ab33405, were flanked by mobile genetic elements, suggesting that they belonged to the pool of genes acquired by HGT (Fig. 2A–D). In addition to the *bla*_OXA-65_, the presence of the *bla*_OXA-23_ was identified and was located within the transposon Tn2008 (Fig. 2A). The *bla*_OXA-23_ has a worldwide distribution, and has been found located in the bacterial chromosome, as well as, in plasmids within a variety of genetic platforms^16,31^. The most prevalent genetic platforms are the Tn2006 and Tn2008 composite transposons^32,33^. These transposons were reported to be associated with the TnAbaR islands, in particular with the TnAbaR4-like island^33^. However, the Tn2008 present in Ab33405 was located outside the TnAbaR-like element. Furthermore, a β-lactamase class A, *bla*_TEM-183_ (*bla*_TEM-1-like_) was identified within the Tn3 transposon. *bla*_TEM-183_ is commonly linked with the Tn1, Tn2 and Tn3 transposons^34^. However, until now, *bla*_TEM-1-like_ in *A*. *baumannii* has been described only as part of the TnAbaR-like element with the presence of fragments of different transposons^16,35,36^. The *bla*_TEM-1-like_ genetic location found in Ab33405 constitutes the first report of this β-lactamase within the complete Tn3 structure in *A*. *baumannii* (Fig. 2B).

Acquired antibiotic resistance against aminoglycoside has been extensively characterized in *A*. *baumannii*^7,37,38^. The presences of five genes (*aadA1*, *sat2*, *strA*, *strB* and *aphA6*) associated with aminoglycoside resistance were detected in Ab33405 genome. In agreement with most of the previous reports, the *aphA6* was found associated within the transposon Tn*AphA6* in the chromosome of Ab33405 (Fig. 2C,D).

Furthermore, we have identified the presence of *aadA1* and *sat2*, conferring resistance to spectinomycin and streptomycin, located within the variable region of the class 2 integrons, embedded in the Tn7 transposon (Tn7::In2-7). In addition, the gene cassette *dfrA1* and the genes *orfX*, *ybfA*, *ybfB* and *ybgA* were also present. The presence of Tn7::In2-7 in Ab33405 (CC 79/113) is in agreement with Ramirez *et al*.’s report, showing a high prevalence of the class 2 integron platform within CC79/113^39^ (Fig. 2A). Tn7::In2-7 was also identified in other bacterial species such as *Escherichia coli*, *Pseudomonas* spp, *Shewanella putrefaciens*, *Raoultella terrígena*, *Citrobacter freundii*, among others^40,41^.

The *strA* and *strB* streptomycin resistance genes identified in Ab33405 were linked to a genetic structure containing *floR*, conferring resistance to florfenicol, flanked upstream by ΔIS*CR2* and IS1006, and downstream by IS*CR2* and *sul2*. The *strA*, *strB*, *floR* and *sul2* genes have been frequently associated with various genetic structures present in different bacterial species^33,42,43^. These genes were mostly flanked by different ISs, such as IS26, IS*Aba1*, IS*CR2*, etc.^33,42–44^. Particularly, these genes were found located within the TnAbaR genomic island or within plasmids^14,42,44,45^. In Argentina, the dispersion of the ΔIS*CR2*-*tet*(B)-*tet*(R)-IS*CR2* genetic platform has been reported in XDR-*A*. *baumannii* strains^44^. These structures have been observed in 85% of the isolates as well as in 4 strains reported in the GenBank database (ZW85-1, BJAB0715, A91, 13205)^14,44^. The genetic structure found in Ab33405 represents a new array not previously observed in *A*. *baumannii* genomes (Fig. 2D). Nevertheless, this genetic structure has been identified in a *P*. *mirabilis* that also possesses Tn2008 downstream^46^.

Among the *A*. *baumannii* efflux pumps, we observed that the AdeIJK codifying genes were found in Ab33405 genome. However, genes codifying for the efflux pump AdeABC and its two-component system *adeRS* genes were not identified. Our results are in agreement with Lopez *et al*.’s that showed the absence of the AdeABC efflux pump and its regulatory system in the Ab421 strain, which belongs to the ST79^47^.

### Potential virulence traits and secondary metabolites in Ab33405

Lately, studies have been focusing on virulence factors in the genus *Acinetobacter*^48,49^. Genomic approaches, together with phenotypic assays and infection models, have contributed in the identification of important virulence factors for *A*. *baumannii* that play a role in pathogenicity^49^. Virulence factors involved in adhesion and motility (e. g. *ompA*), biofilms and its regulation (e.g. Bap, Flagelum Csu, PNAG, BfmSR), evasion of the immune system (e. g. capsule, LPS), and iron uptake (e. g. acinetobactin)^7,49,50^ were studied.

The Ab33405 genome was examined for genes coding for potential virulence factors using VFDB database. A total of 85 gene hits were found which facilitate the identification, evaluation, and validation of virulence factors in this strain.

To identify virulence genes among the 85 gene hits found, we focused our analysis on well-characterized determinants.

There is vast evidence that adherence to the host cell represents the initial stage of colonization and/or infection. During colonization, bacteria can form microcolonies giving rise to a highly structured microbial community, called biofilm^49,51^. The initial stage of biofilm formation in *A*. *baumannii* is mediated by the *CsuA/BABCDE* operon coding for a fimbriae chaperone, which plays a key role for the assembly and production of pili involved in surface adhesion^52^. A two-component system (*bfmSR*), including the kinase sensor encoded by the *bfmS* and the response regulator encoded by the *bfmR*, was reported to be involved in the regulation of *CsuA/BABCDE* operon^51^. Moreover, for the development of the mature structure of biofilms, an ortholog of the biofilm-associated protein Bap of *Staphylococcus* spp. has been identified and was first reported in *A*. *baumannii* AB307-0294 strain^53^. We confirmed the presence of the *CsuA/BABCDE* operon and the *bfmSR* system in Ab33405 genome, as well as *bap*.

Additional virulence factors that have been vastly studied are the iron uptake systems, which are known to be involved in survival under iron-limiting conditions^21,49,54^. When *A*. *baumannii* encounters an iron-limiting environment, it can produce the acinetobactin siderophore^54,55^. The locus responsible for the synthesis, utilization, and secretion of acinetobactin (also called *acinetobactin* locus), is composed of eighteen genes. Within this gene group, ten *bas* genes are responsible for siderophore synthesis, six *bau* genes for the use of acinetobactin, and two *bar* genes for acinetobactin secretion. Furthermore, ferric acinetobactin is transported into the bacterial cells with the help of specific outer membrane receptors (BauA), periplasmic proteins (BauB, TonB, ExbB, ExbD) and internal membrane associated proteins (BauCDE)^21,54,55^. Once internalized, the ferric-siderophore complexes are reduced to release iron by an enzyme with ferric reductase activity (BauF). Penwell *et al*.^56^, showed that the *entA* and *entB* genes, as well as *bas* genes, are necessary for the synthesis of the acinetobactin precursor, which is found outside the acinetobactin locus previously mentioned^56^. However, Hasan *et al*.^57^, reported the existence of variability in the gene content of the acinetobactin locus, as well as in the *entA* and *entB* (*entAB*) genes, suggesting HGT as a mechanism in the acquisition of these genes^57^. Analysis of the Ab33405 genome showed the presence of the acinetobactin locus and *entAB* locus reported for the *A*. *baumannii* ATCC 19606^55,56^. TonB, a protein also related with iron uptake, is responsible for the transduction of signals that allows the transport of iron. Three different copies of *tonB* were reported in the literature, of which *tonB1* and *tonB3* are presented in a structure forming an operon with the *exbB* and *exbD* genes, while *tonB2* is found in monocistronic form^58^. The three copies of *tonB* reported by Zimbler *et al*. were identified in the Ab33405 genome. The acinetobactin locus, the *entAB* genes, as well as the *tonB* genes were identified in Ab33405 genome, which led us to infer that Ab33405 would be capable of producing acinetobactin and capturing host iron for survival.

Penwell *et al*.^59^, reported the capacity of some *A*. *baumannii* strains producing an alternative iron chelating molecule^59^. This alternative siderophore, called baumannoferrin, was characterized in the AYE strain, which is deficient in the production of acinetobactin^56,59^. The baumannoferrin siderophore has a higher affinity for iron than acinetobactin, and the internalization of ferric baumannoferrin is independent of the machinery encoded by the *bau* genes. The baumannoferrin (*bfn*) locus responsible for baumannoferrin biosynthesis is composed of 12 genes (*bfnA-L*) that encode proteins similar to those involved in the production, use, and transport of acinetoferrin siderophore and achromobactin^59^. We also identified the presence of the *bfn* locus in the Ab33405 genome. Considering the similarity of the Bfn proteins with those described in other species of *Acinetobacter* and *Pseudomonas*, and with the knowledge that the *bfn* locus is not present in all *A*. *baumannii* strains, we would suggest that this locus was acquired through HGT (see Supplementary Fig. S4).

**Figure 3:**
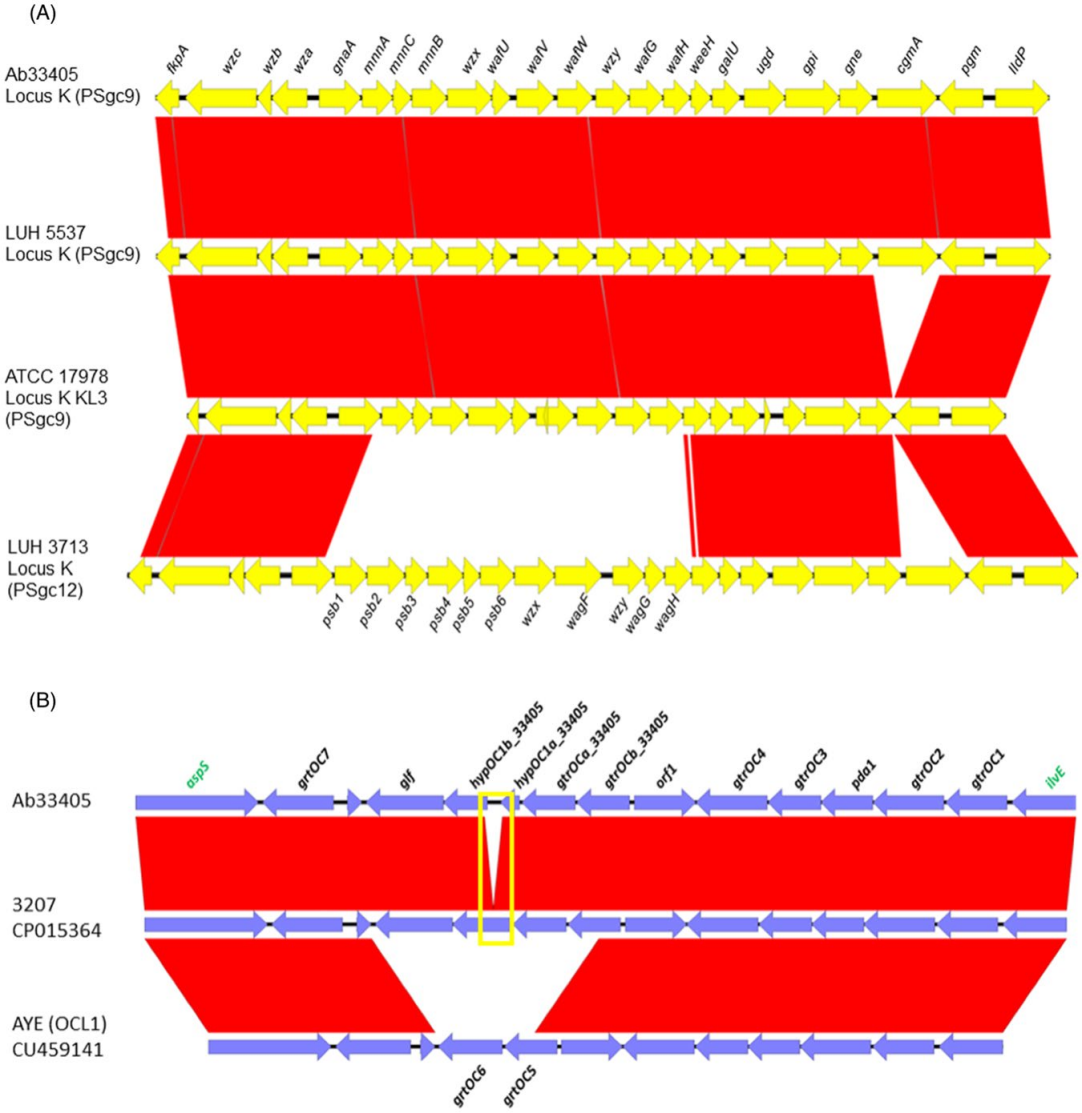
Comparison and genetic structure of K locus and OC locus of *A*. *baumannii* Ab33405 strain. (**A**) The K locus genes is indicated in black and flanked genes are indicated in green. (**B**) The OC locus is indicated in black and flanked genes are indicated in green. Both locus structure, K and O, were compared to the most similar structures present in the GenBank databases. The yellow square indicates the 226-nucleotide difference between Ab33405 and 3207 strains. The graphic representation was made using the EasyFig. 2.2.2 software.

The capsular polysaccharide is considered an important virulence trait in Gram-negative bacteria allowing bacteria to resist the bactericidal activity of the complement. In Gram-negative bacteria, capsular polysaccharide biosynthesis loci (KL, K locus) and LPS loci (OCL, OC locus) are typically “hot regions” of genomic variability. Kenyon *et al*. 2013, identified 9 KL and 3 OCL^60^ in ten analyzed genomes of *A*. *baumannii*. A contemporary work performed by Hu *et al*. 2013, included 217*A*. *baumannii* genomes and showed the presence of 77 varieties of KL (or locus K), which were named as PSgc^61^. Analysis of Ab33405 genome allowed us to identify the presence of both OCL and KL in the genome. Analysis and comparison of the overall structure of KL for Ab33405, with a GC content percentage of 33.35%, showed 99% nucleotide identity and 100% coverage with the strain Ab LUH5537 (PSgc9) described by Hu *et al*.^61^ (Fig. 3A). This structure has a high similarity with the locus KL3 present in the reference isolate *A*. *baumannii* ATCC 17978, which has been described and denominated as KL3 according to the classification proposed by Kenyon *et al*.^60^ (Fig. 3A). The KL present in Ab33405 contains the *cgmA* gene, which is absent in the KL3 locus described in strain ATCC 17978 (Fig. 3A).

Furthermore, we also characterized the OCL, which is responsible for the synthesis of the O antigen, demonstrating the variability present in the genome of Ab33405. The OCL of Ab33405, with a GC content of 36.01%, showed similarity with genetic structures found in the genomes of AbH12O-A2, AB030 and 3207 (Fig. 3B). The difference between *A*. *baumannii* Ab33405 and 3207 is 226 nucleotides, denoted with a yellow square in Fig. 3B. Among the OC locus reported by Kenyon *et al*. 2013, the most similar genetic structure is the OC OCL1 locus of *A*. *baumannii* AYE (CU459141)^60^ (Fig. 3B). Therefore, it can be inferred that the Ab33405 strain, together with the 3207 strain, presents a new OC locus. According to the classification made by Kenyon *et al*. 2014, the OC locus related to OCL1 would belong to group A.

**Figure 4:**
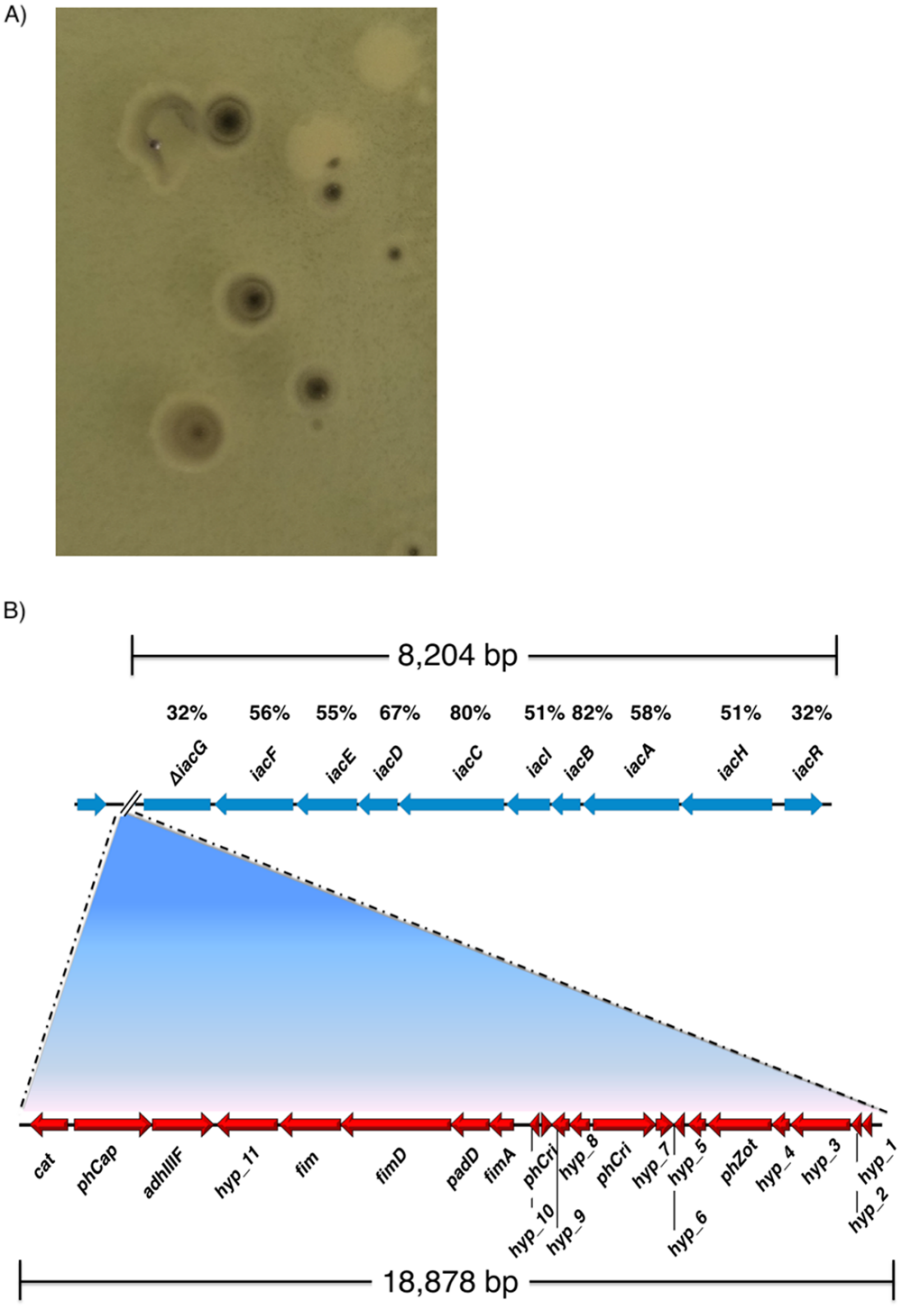
Comparison and genetic organization of *iac* locus on *A*. *baumannii* Ab33405. (**A**) Expression of the indigo-pigmented phenotype by *iacA* of *A*. *baumannii* Ab33405 cloned in pIACA into *E*. *coli* TOP10. (**B**) Schematic representation of the *iac* locus and its comparison to the *iac* locus present in *Pseudomonas putida* 1290. The insertion into the *iacG* gene is shown. Percentages represent the amino acid identity with the *iac* locus described in *P*. *putida* 1290 strain.

*A*. *baumannii* Ab33405 produces a characteristic indigo pigment when cultured in SIM medium (sulfide indole medium for mobility). This pigment is similar to that synthesized by *Pseudomonas putida* 1290 by oxidation of indole-3-acetic acid (IAA)^62^. This process has been associated to the *iac* locus, which includes the *iacA* gene. This gene codes for the indole-oxygenase that catalyzes the oxidation reaction. As it is the case for other *A*. *baumannii* strains like ATCC 17978 and ATCC 19606^63,64^, strain Ab33405 possesses a homolog to the *P*. *putida* 1290 *iac* locus. However, no *A*. *baumannii* strain had been described that produces the pigment when cultured in the presence of indole-3-acetic acid^63^. To confirm that *iacA* is responsible for the production of the pigment, we cloned this gene and introduced the recombinant plasmid, pIACA, into *E*. *coli* TOP10. Plating of the transformed strain resulted in pigmented colonies (Fig. 4A). This result led us to hypothesize that the unique property of strain Ab33405 is due to inactivation of *iacG*, which codes for a putative repressor, by insertion of a phage genome (Fig. 4B).

**Figure 5:**
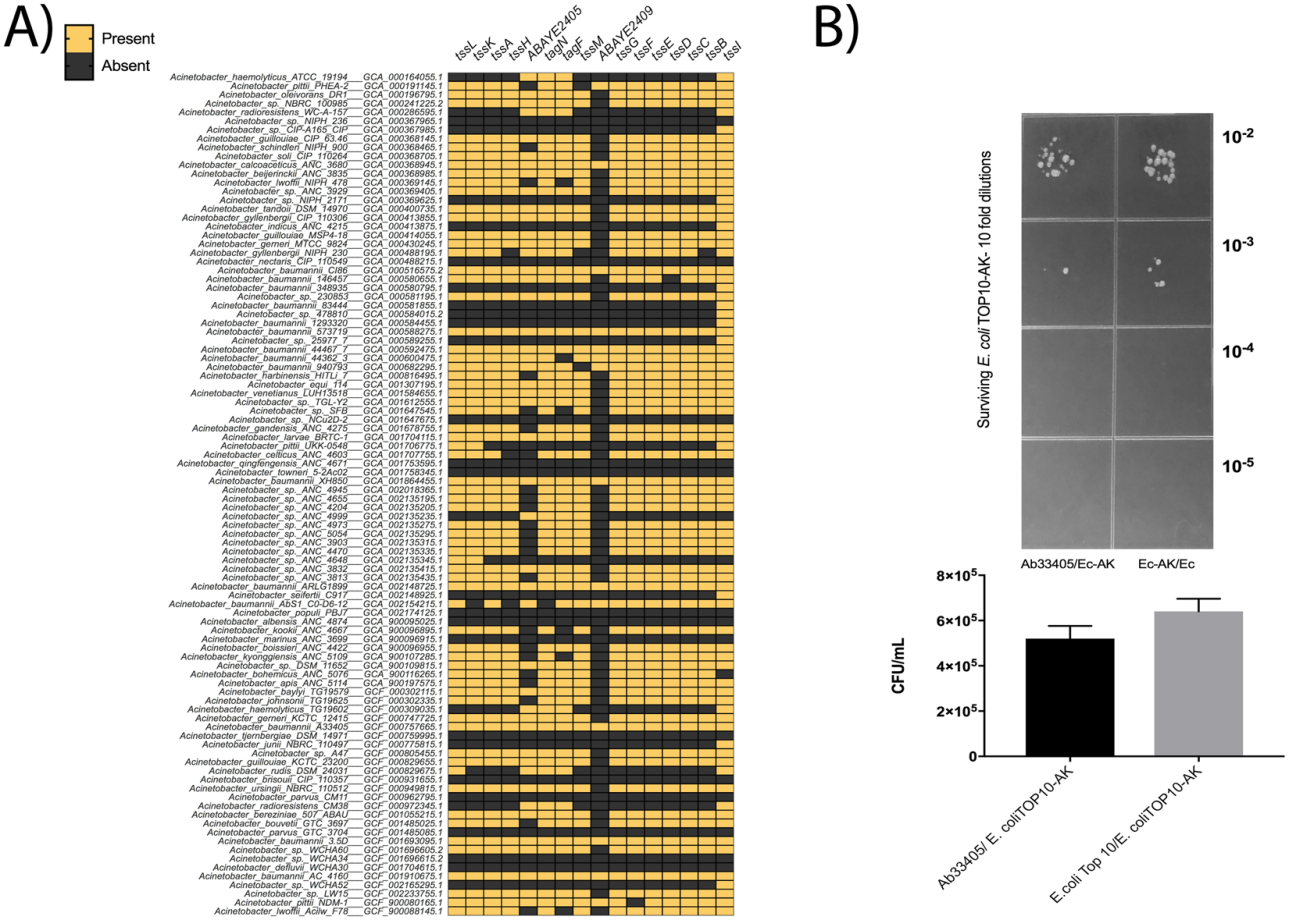
Genomic comparison of genes coding for T6SS system. (**A**) Distribution of the T6SS components in *Acinetobacter*. Each component of the T6SS was mapped on the 96 sampled genomes, representatives of the diversity of the genus. Genes were searched by means of BLASTP, using a maximum e-value of 1^e-5^, a minimum identity value of 50%, and query coverage of 75% as threshold. The genes of *A*. *baumannii* AYE were used as query in all cases, as defined in Weber et al. 2013. Orange boxes show presence and gray boxes show absence of T6SS genes. The heatmap was generated using GraphPad Prism version 7 (**B**) Bacterial Killing assay using *E*. *coli* TOP10-AK (Prey) and Ab33405 (Predator). Representative LB-AK agar plate showed differences in survival of bacterial colonies. The experiment was performed two times.

*A*. *baumannii* strains possess a T6SS that has the ability to mediate killing of bacterial competitors. This system consists of thirteen core proteins and numerous accessory proteins^48^. The thirteen core proteins are present in the Ab33405 genome (Fig. 5A). A general analysis showed wide variability in the presence or absence of the T6SS core genes in *Acinetobacter* species. This study expands and agrees with a previous analysis that found high heterogeneity in the presence of T6SS components in *A*. *baumannii*, *A*. *calcoaceticus*, *A*. *oleivorans*, *A*. *baylyi*, *A*. *johnsonii*, *A*. *radioresistens*, and *A*. *lwoffii* strains^48^. It is possible that the high variability in the content of T6SS genes reflects an adaptation to the hostility of the environment.

The ability of Ab33405 to kill competitor bacteria was tested against *E*. *coli* cells^65,66^. Figure 5B shows that Ab33405 killed the *E*. *coli* cells suggesting an active T6SS.

The genus *Acinetobacter*, and in particular *A*. *baumannii*, has been shown to sense and respond to light^67–71^. While many BLUF type photoreceptors are present in non-*baumannii* species, the presence of only one BLUF protein has been detected in *A*. *baumannii*, designated BlsA. Recently our group showed that susceptibility to certain antibiotics such as minocycline (MIN) and tigecycline (TIG), has also been shown to be modulated by light by a mechanism independent of BlsA which likely relies on singlet oxygen^67^.

**Figure 6:**
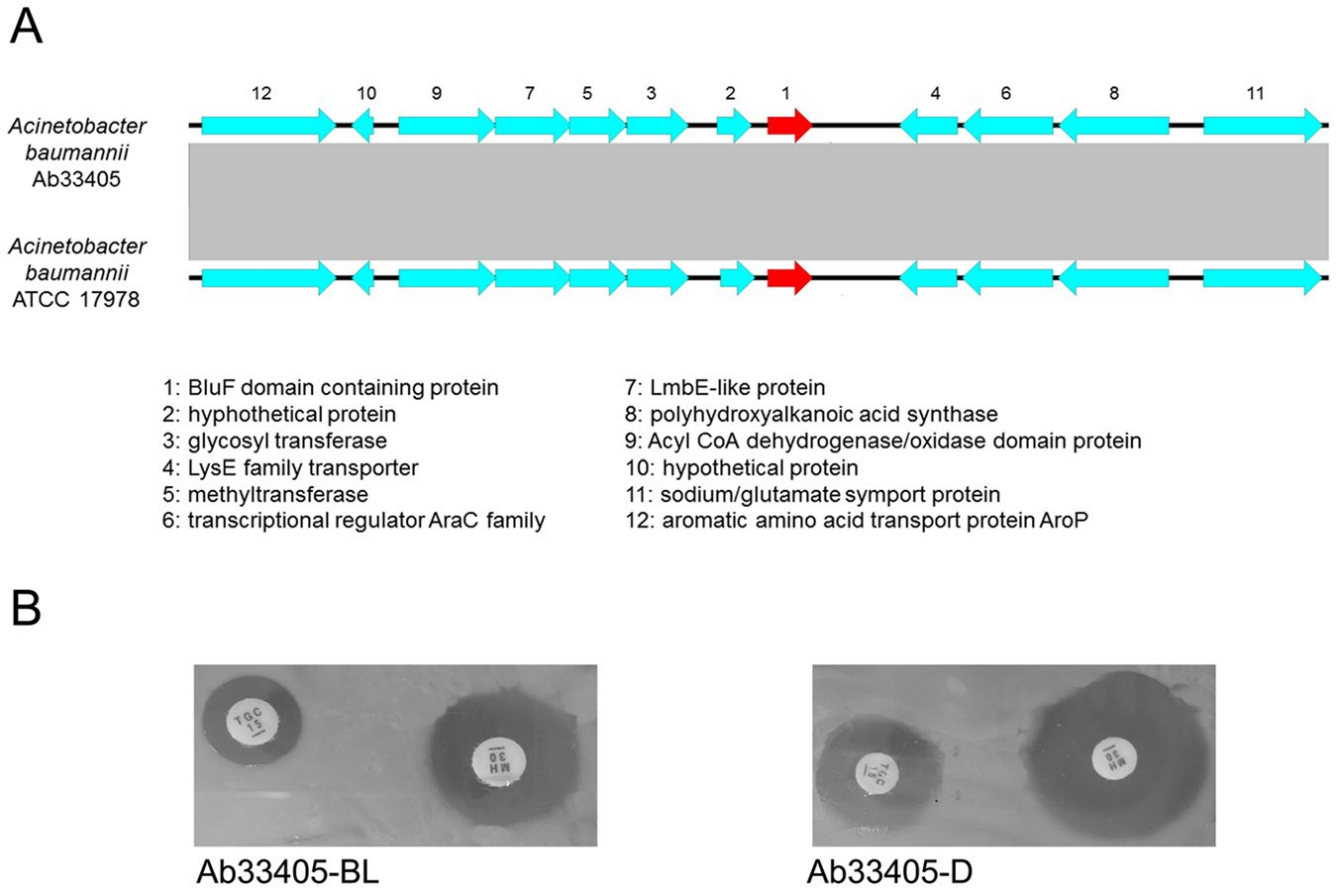
(**A**) Schematic representation of the genetic environment of *bls*A in Ab33405 genome. *blsA* is represented in red. (**B**) Disk diffusion agar plates of minocycline 30 mg (MH) and tigecycline 15 mg (TGC). Plates were photographed after incubation overnight in darkness (**D**) or in the presence of blue light (BL) at 24 °C. The experiments were repeated at least three times for each condition.

Analysis of the Ab33405 genome reveals the presence of only one putative photoreceptor, which belongs to the BLUF type, as is the case in other *A*. *baumannii* strains previously described^68,69^. This BlsA homolog is 98% identical to the one present in strain *A*. *baumannii* ATCC 17978, and 99% identical to the homolog present in strain *A*. *baumannii* ATCC 19606. Moreover, the genomic region containing *blsA* in Ab33405 is conserved with respect to *A*. *baumannii* ATCC 17978, showing 98% identity in the 6,000 bp each side flanking *blsA* (Fig. 6A). In these strains, *blsA* is flanked on one side by proteins homologous to a LysE family transporter, a transcriptional regulator of the AraC family, a polyhydroxyalkanoic acid synthase, and a sodium/glutamate symport protein. On the other side, BlsA is flanked by proteins homologous to an oligosaccharide/oligonucleotide binding protein (BOF), a glycosyltransferase, a methyltransferase, a LmbE-like protein and an Acyl-CoA dehydrogenase/oxidase.

To test the ability of Ab33405 to sense and modify its response to antibiotics under light conditions, susceptibility assays under light or dark conditions were performed. It was observed that Ab33405 can modulate susceptibility to MIN and TIG. An increase in the halo of inhibition from 18 mm to 24 mm (∆6) and from 12 mm to 18 mm (∆6) were observed when Ab33405 was incubated under dark conditions at 24 °C for MIN and TIG, respectively (Fig. 6B).

### Other mobile elements not linked with resistance determinants in Ab33405 genome

We have identified the presence of mobile genetic elements, such as transposons, ISs, phage sequences, and genomic islands (GIs) in the Ab33405 genome. We determined the presence of fourteen GIs, eight putative prophages, seven ISs and three transposons. The distribution of the mobile elements was shown along the entire genome topology, and it was not localized into a specific region on Ab33405 genome. Moreover, we have identified the association of phages sequences and ISs to GIs (Fig. 1).

As we mentioned above eight complete ISs were identified in Ab33405 genome (Fig. 1). Among the ISs identified we found IS*Aba1*, IS1006, IS*Aba31*, two copies of IS*Aba125*, IS26, and IS*Aba39* and IS*CR2*.

We decided to explore the genetic context of the ISs present in Ab33405. The genetic context of IS*Aba1*, IS*Aba125*, IS1006 and IS*CR2* were mentioned above, as they were linked with antibiotic resistance determinants.

**Figure 7:**
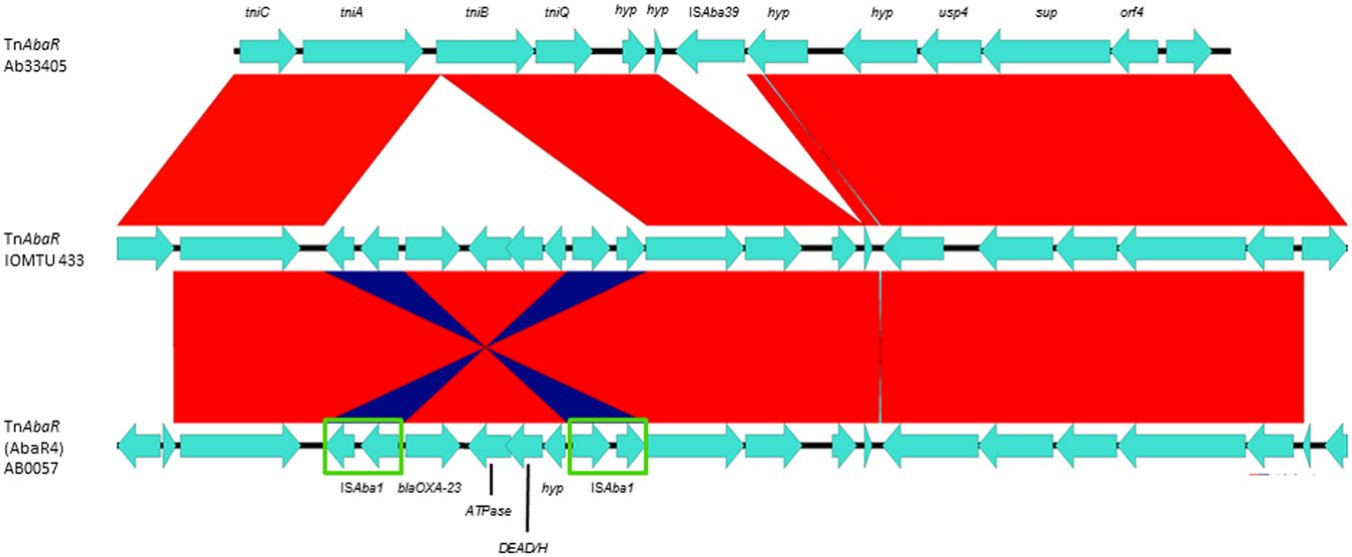
Comparison and genetic organization of the TnAbaR-like genetic island within Ab33405 genome. The Ab33405 TnAbaR-like genomic island was compared against to the most similar TnAbaR-like genetic structures that were found in the GenBank database. Red links represent homolog sequences and blue links represented inversions. The graphic representation was made using the EasyFig. 2.2.2 software.

Here, we described the context of the other ISs: (i) IS*Aba31* was close to a gene that codifies for a lipase and to *tonB* (see Supplementary Fig S5A), (ii) IS26 was disrupting an oxidoreductase gene that was located upstream of a putative lipoprotein (see Supplementary Fig S5B), and (iii) IS*Aba39* was found within the AbaR-like island. IS*Aba39* (also named as IS*Aba42*) is as a new IS recently described that belongs to IS256 family, which possess the corresponding inverted repeats and directed repeats related to IS256 family (Fig. 7). Furthermore, we did the sequence comparison with ten transposases of IS256 family and we found that the highest similarity was with a transposase described within a plasmid (pACI-3bd5) found in the *Acinetobacter* sp. ACNIH1. Also, IS*Aba39*(IS*Aba42*) possess 86% and 69% amino acid identity with IS*Ec39* and IS*Aba26*, respectively.

**Figure 8:**
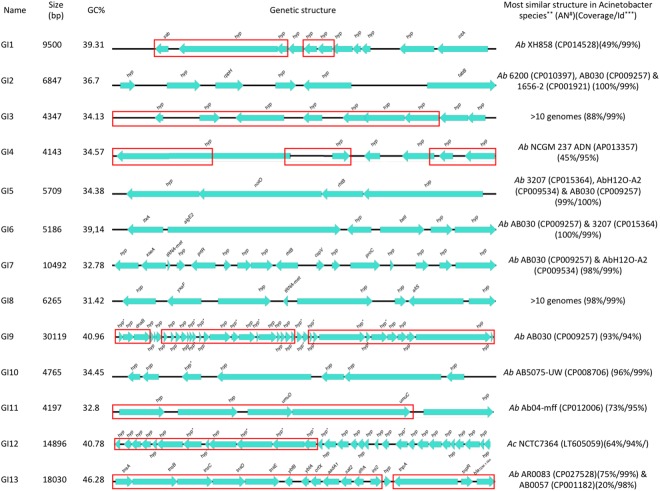
Genetic structure of genomic island within Ab33405. The red box represented the partial coverage region against to the most similar genetic structure in other *Acinetobacter* genome. The (*) represented the presence of phage sequences. The graphic representation was made using the EasyFig. 2.2.2 software.

A total of fourteen GIs, named as GI1 to GI14, were found in Ab33405 genome. Thirteen were identified using the IslandViewer3 tool and the TnAbaR-like island was found by additional bioinformatic analysis (Figs 1 and 8). The sequences of the predicted GIs were analyzed in detail, and we observed that eleven of the thirteen (84.61%) GIs predicted by IslandViewer3 contained prophage sequences in their structure (Figs 1 and 8). We also noted that the complete genetic structure of five of the thirteen (38, 5%) predicted GIs were also present in other *A*. *baumannii* isolates as well as other species within the genus. The rest of the GIs showed variable alignment coverage (45–98%) with a high nucleotide identity (98–100%). Interestingly, the predicted GI13 corresponds to the region mentioned in the section describing the antimicrobial resistance determinants. This GI, which contains *dfrA1*, *sat2* and *bla*_TEM-183_, and the mobile elements, Tn7::In2–7 and Tn3, could be considered a novel resistance island, not previously described in *A*. *baumannii* (Fig. 8). Furthermore, using the GenBank database, the genetic structure of GIs was searched in other bacterial isolates in order to show their distribution. The size, GC%, genetic structure and similar structure previously described are illustrated in Fig. 8.

The TnAbaR present in Ab33405 was not previously described. It has a size of 14,821 bp and resembles the ones described in *A*. *baumannii* IOMTU 433 and TnAbaR4 island of *A*. *baumannii* AB0057 (Fig. 1). This novel structure contains the new IS*Aba39* (Fig. 7). Although, the similarities observed between the Ab33405′s TnAbaR-like and TnAbaR4, the absence of *bla*_OXA-23_ within the context of the island was observed in Ab33405. The lack of resistance genes on Ab33405′s TnAbaR demonstrates that acquired antibiotic resistance would not necessarily be restricted to a particular region.

PHAST database was used to identify phage sequences in *A*. *baumannii* Ab33405 genome. This analysis identified the presence of eight regions related to phage sequences of which four regions were classified as “intact,” one region as “incomplete” and the three regions as “questionable” (Fig. 1). The complete sequences of the eight predicted phages found in our strain were unique. No identity was found with any of the available sequences deposited in the GenBank to date. However, partial phage sequences were detected in several *A*. *baumannii* genomes.

Our analysis showed that within three of the structures (region 2, 3, and 5) predicted to be “intact”, the presence of the integrase as well as its specific recombination sites *att* were found. However, the region 4 that was categorized as an “intact” phage by PHAST, did not present integrase or the site-specific recombination sequence *att*. We observed a linkage of two such prophages to the genus *Acinetobacter* (region 2 and 5), while the other two were linked to the genera *Psychrobacter* (region 3) and *Burkholderia* (region 4). The predicted phage in region 2 has two *att*L sites (*att*L1: AACTGAAGTTAA and *att*L2: AATTTATAAAAT) and two *att*R sites (*att*R1: AATTTATAAAAT and *att*R2: AACTGAAGTTAA). The coding sequences within the genetic structure of the prophage are composed mostly of hypothetical proteins. The region 3 presents an *att*L site (CGCTCTAAATTGAGCGCTTTTT) and an *att*R site (CGCTCTAAATTGAGCGCTTTTT). As mentioned before, region 5 presented the integrase and two *att*L (TTCAGGCTCAAAA) and *att*R (TTCAGGCTCAAAA). Among its non-essential proteins, we mainly found hypothetical proteins and a transfer RNA (tRNA) (see Supplementary Fig S6).

Sequence analysis of the prophages that were predicted in the questionable (region 1, 7, and 8) and incomplete (region 6) category have been described in the genus *Acinetobacter*. By analyzing the questionable prophages, we noted the site-specific recombination sites *att* and the coding sequence for integrase in two of the predicted prophages (Region 1 and 8). Region 1 showed two site-specific recombination sites, *att*L (TAATTTTTTTCA) and *att*R (TAATTTTTTTCA), the presence of an integrase, and also possessed non-essential genes, such as genes coding for hypothetical proteins and phosphoethanolaminatransferase associated with resistance to polymyxin. Region 8 presents two site-specific recombination sites, *att*L (ACATCATGCCTAAC) and *att*R (ACATCATGCCTAAC), the presence of an integrase and non-essential genes coding for hypothetical proteins, zinc dependent hydrolase, and a diacylglycerol kinase (see Supplementary Fig S6). Finally, region 6, that was predicted as an incomplete prophage, showed the presence of an integrase and two site-specific recombination sites, *att*L (ATGCTTCTAATGATCGA) and *att*R (ATGCTTCTAATGATCGA) (see Supplementary Fig S6).

## Conclusion

Genomic comparison of this pigmented *A*. *baumannii* strain revealed the presence of a variety of sequences not previously described in this species, reinforcing the idea of the heterogeneity in the content and organization of *A*. *baumannii* genomes. Genes not previously described in this species, a significant amount of mobile genetic elements, as well as different genetic structures related to antibiotic resistance and virulence traits, were found in Ab33405. The *iac* locus presented in Ab33405 had an insertion of 18 kb into the *iacG* gene that could explain the uncommon pigmentation observed in this strain. Taken together our results clearly showed the versatility of *A*. *baumannii* to remodel its genome. Our data adds more shreds of evidence of how *A*. *baumannii* can evolve and adapt through various sources of genetic variability. Such genetic variability would generate adaptation and rapid evolution in the hostile situations that are faced in the hospital environment.

## Material and Methods

Fig. S7 represents the work pipeline describing all the procedures used in the present study (see Supplementary Fig. S7).

### Bacterial isolate

The *A*. *baumannii* clinical isolate Ab33405 was previously reported as part of an indigo-pigmented outbreak^14^. This strain was isolated from a 65 year-old male patient from the coronary care unit. The strain belongs to the.

CC113^B^/CC79^P^ clonal complex^14,72^, which was shown to be a prevalent clonal complex in clinical *A*. *baumannii* isolates from Argentina^73^. It was categorized as extensively drug-resistant (XDR) according to the recent definitions suggested by Magiorakos *et al*.^74^ as it is susceptible to only amikacin, colistin, tigecycline and minocycline and resistant to ceftazdime, cefepime, piperacillin-tazobactam, imipenem, meropenem, gentamicin, trimethoprim- sulfamethoxazole and ciprofloxacin^14^.

### General molecular techniques, DNA sequencing and assembly

Total DNA was obtained using the Master Pure DNA purification kit by following manufacturer’s instructions (Epicentre, Madison, WI, USA). Ab33405 was sequenced using Illumina MiSeq at the Argentinian Consortium of Genomic Technology (ACGT). De novo assembly was performed with SPADES assembler version 3.1.0^75,76^, using a pre-assembly approach with Velvet^76^.

### Cloning of iacA gene from Ab33405 in *E. coli*

The *iacA* was cloned in pCR™2.1-TOPO® vector using the TOPO® TA Cloning® Kit from ThermoFisher Scientific (Waltham, MA USA). Positive clones, were confirmed by sequencing (pIACA). *E*. *coli* TOP10 cells were transformed with pIACA and plated on LB agar plates containing kanamycin 20 µg/ml, incubated at 37° for 18 hrs, and then left at room temperature for at least 48 hrs to observe the production of pigmentation in *E*. *coli* strain (Fig. 4B).

### Sampling method for phylogenetic analyses, identification of homologous genes, and phylogenetic tree reconstruction

All genome assemblies and protein coding sequences of the genus *Acinetobacter* were downloaded via ftp from NCBI website (August 8th, 2017). This first dataset comprised 2895 assembled genomes. In order to avoid over-represented species, genomes were sampled based on the sequence of forty-nine highly conserved ribosomal protein-coding genes (see Supplementary Table S4). All these genes were identified in 2545 genomes by means of PSI-BLAST searches^77^, best hits were considered positive if the identity value was higher than 50% and the query coverage was above 75%. These 2545 genomes were used for further analysis (see Supplementary Table S3). The 49 ribosomal protein coding genes from the 2545 genomes were independently aligned using MUSCLE^78^ with the fastest algorithm (options: -maxiters 1 -diags1). Quality trimming of alignments were done using GBLOCK software with default parameters^79^. Trimmed alignments were subsequently concatenated for sequence distance estimation. Strains displaying a 99% or more similarity at the amino acid level in these conserved genes were clustered and considered genetically equivalent. One strain for each cluster was randomly selected for subsequent phylogenetic analysis with preference for those with assigned species name, if available. The resulting second dataset comprise 95 selected genomes, assumed as representatives of the genetic diversity of the genus, plus the genome of Ab33405 (see Supplementary Table S3).

Putative homologous genes among genomes of this second database were identified by means of the GET_HOMOLOGUES software^80^ using the OrthoMCL method^81^. BLASTP searches were performed with a maximum e-value of 1^e-5^, a minimal identity value of 30%, and a minimal query coverage of 75%. Six hundred fifty-six putative orthologous genes were identified among the 96 genomes analyzed and used for phylogenetic analysis. Coding sequences were translated into proteins by means of the *transeq* program implemented in the EMBOSS package^82^. The protein sequences were aligned using CLUSTAL OMEGA^83^. Phylogenetic trees were inferred using the maximum-likelihood method with an amino acid LG + G model by means of PHYML version 3.0^84^. The default SH-like test was used to evaluate branch supports^85^. Finally, a consensus tree was inferred from the 656 phylograms using the SUMTREES.PY program from the DENDROPY package^86^.

The pan-genome, the soft-core genome, and the core-genome of the genus *Acinetobacter* were identified using the GET_HOMOLOGUES software^80^ based on the sampled 96 genomes.

A second phylogram was built in order to get a more detailed picture of the evolutionary relationships within the *Acinetobacter calcoaceticus–baumannii* complex. For this phylogenetic analysis a second approach, based on SNPs identification, was implemented. Computational less expensive methods were used, considering the number of sequences and the expected genetic distance of the genomes. In brief, phylogenetically related genomes to Ab33405, which are clustered in the same significant monophyletic group (see Supplementary Fig. S2, indicated with a green box), were used to track back all closely related genomes to the original first local dataset. As a result, 2293 genomes were retrieved (see Supplementary Table S3). 164 highly conserved genes were identified among these genomes within the original set of putative orthologs by means of psi-blast (see Supplementary Table S3). Sequences were aligned using MUSCLE^78^ (options: -maxiters 1 -diags1) and trimmed using GBLOCK^79^. Generated blocks from all alignments were finally concatenated and clustered, completely identical sequences were clustered (100% identity). Again only one sequence from each cluster was selected, comprising 1037 closely related strains plus Ab33405. Variable aligned positions were extracted using SNP-SITES^87^ and a maximum likelihood phylogenetic tree was built using RAXML 8.2.9 using the ASC parameter for variable sites and the RELL bootstrap technique for node support assessment (see Supplementary Fig S8).

### Gene prediction and comparative genomics

Gene prediction and annotation were performed using RAST server^88^. Contigs were ordered and oriented using multiple references by means of RAGOUT software with default settings^89^. The resulting scaffolds, comprising a total of 3675699 bp., the assembled genome sequence, annotation, and scaffolding are available at www.higiene.edu.uy/ddbp/Andres/gtraglia_et_al_2018b_data.html. Visualization of genome comparison with these genomes was done using BRIGS application^90^. Complete genomes of phylogenetically closely related strains were used as reference genomes for scaffolding the Ab33405 assembly (blue box in Supplementary Fig. S3). Selected strains were: AF401 (Acc. Num. GCA_001896005.1), 3207 (Acc. Num. GCA_001636235.1), AbH12O-A2 (Acc. Num. GCA_000761175.1), AB030 (Acc. Num. GCA_000746645.1), 11510 (Acc. Num. GCF_001922425.1). Strain ACICU (Acc. Num. GCA_000018445.1) was also included as reference sequence. Contigs longer than 1000 bp were used to estimate the average nucleotide identity (ANI) between genomes^91^ by means of the ani.rb script, while average amino acid identity was estimated using aai.rb. Both scripts were developed by Luis M. Rodriguez-R and are available at (enveomics.blogspot.com).

The virulence factor genes were predicted using the virulence factor database (VFDB)^92^. The mobile genetic elements were identified using the tRNAscan-SE for the tRNA gene- related genomic islands (GI)^93^, PHAST for phages and prophages^94^, and ISFinder for insertion sequence (IS) elements^95^. Moreover, antimicrobial resistance genes were predicted by ARG-ANNOT^96^.The presence and genetic environment of *blsA* gene was performed by BLAST^97^.

### Killing Assay

These assays were performed using Ab33405, *E*. *coli* TOP10-AK, and *E*. *coli* TOP10 as predator and prey, respectively, as recently described^65,66^. Ab33405, *E*. *coli* TOP10-AK and *E*. *coli* TOP10 cultures were grown until they reached an OD_600_ = 0.5, washed, and resuspended in 1 ml of saline solution. Equal volumes of prey and predator cultures were mixed and 10 μl were spotted on LB agar plates. After a 4-h incubation at 37 °C, the bacterial spots were resuspended in 1 ml of LB and 10 μl of 10-fold serial dilutions were spotted on LB agar containing 50 μg/ml amikacin. *E*. *coli* TOP10-AK/*E*. *coli* TOP10 mixtures were used as negative controls. Bacterial growth on the surface of the plates was recorded after incubation at 37 °C for 36-h. Killing assays were repeated twice using independent biological samples each time^65,66^.

### Disc diffusion antibiotic susceptibility test under light and dark conditions

Antibiotic susceptibility assays were performed following with the procedures recommended by the CLSI with slight modifications as described in Ramirez *et al*.^67^. Luria Bertani (LB; Difco) agar plates were inoculated with 100 µl of culture of each tested strain, which was previously resuspended in physiologic solution and adjusted to OD_600_ = 0.1. Antimicrobial commercial discs (BBL, Cockeysville, MD, USA) containing 10 mg of ampicillin, 30 mg of amikacin, 30 mg of cefepime, 30 mg of cefotaxime, 30 mg of cefoxitin, 30 mg of cephalotin, 30 mg of chloramphenicol, 5 mg of ciprofloxacin, 10 mg of imipenem, 10 mg of gentamycin, 10 mg of meropenem, 100 mg of piperacillin, 5 mg of rifampicin, 15 mg TIG or 30 mg MIN were placed on the surface of plates, which were later incubated overnight at 24 °C in the dark or under light emitted by nine-LED (light-emitting diode) arrays with an intensity of 6 to 10 µmol photons/m2/s. The assays were performed in triplicate. Breakpoints defined by the CLSI criteria for MIN in MH solid media were considered: susceptible ≥16 mm; intermediate 13–15 mm; resistant ≤12 mm. The breakpoint criteria used to determine the TIG phenotype was based on the United States Food and Drug Administration for *Enterobacteriaceae* and considers susceptibility ≥19 mm; intermediate 15–18 mm; resistant ≤14 mm.

### Data access (AN)

This sequence data of this study has been submitted to the NCBI BioProject (http://www.ncbi.nlm.nih.gov/bioproject) under BioProject accessions: SAMN02951536/JPXZ00000000. In addition, the assembled genome sequence, annotation, and scaffolding are available at http://www.higiene.edu.uy/ddbp/Andres/gtraglia_et_al_2018b_data.html.

## Electronic supplementary material

Additional information

Table S2

Table S3

Table S4

Table S5
