# Supplementary material for: Genome sequence analysis of an extensively drug-resistant Acinetobacter baumannii indigo-pigmented strain depicts evidence of increase genome plasticity
Source: Sci Rep. 2018 Nov 16;8:16961. doi: 10.1038/s41598-018-35377-5 (PMC6240043; doi:10.1038/s41598-018-35377-5)
Supplement: Supplementary file 1 — Additional information [file 41598_2018_35377_MOESM1_ESM.pdf]

Additional Information.

Supplementary information accompanies this paper

Genome sequence analysis of an extensively drug-resistant *Acinetobacter baumannii* indigo-pigmented strain depicts evidence of increase genome plasticity

German Traglia[1], Kevin Chiem[1,2], Brettni Quinn[2], Jennifer S Fernandez[2], Sabrina Montaña[1], Marisa Almuzara[3], María Alejandra Mussi[4], Marcelo Tolmasky[2], Andres Iriarte[5], Daniela Centrón[1], and María Soledad Ramírez [2]***.**

[1]Instituto de Microbiología y Parasitología Médica (IMPaM, UBA-CONICET), Facultad de Medicina, Universidad de Buenos Aires, Argentina, [2]Department of Biological Science, California State University Fullerton, Fullerton, CA, USA, [3]Laboratorio de Bacteriología, Hospital Interzonal de Agudos Eva Perón, San Martín Buenos Aires, Argentina, [4]Centro de Estudios Fotosintéticos y Bioquímicos (CEFOBI- CONICET), Rosario, Argentina,[5] Laboratorio de Biología Computacional, Dpto. de Desarrollo Biotecnológico, Instituto de Higiene, Facultad de Medicina, Universidad de la República, Montevideo, Uruguay.

Additional Information

Supplementary information accompanies this paper

Legends to Supplementary Figure and Tables

Figure S1. Venn Diagram. Pangenome analysis of the genus *Acinetobacter*. Partial view of the generated Venn Diagram, note that the size of each circle represents the relative number of homologous gene families in each category. In the diagram is indicated the estimated number of homologous gene families that **Figure S2. Phylogeny *Acinetobacter* genus**. Inferred phylogenetic relationships within the genus *Acinetobacter*. Ab33405 strain plus 95 genomes, representatives of the genetic diversity of the genus, were included in this analysis. This consensus phylogram was inferred from 656 maximum-likelihood trees, one from each cluster of orthologs. Node support derived from the percentage of phylogenies in which a certain node appeared. The number of conserved homologous gene families between Ab33405 and each selected strains in the phylogeny is indicated. These values were derived from the pangenome matrix generated with get_homologues software. The monophyletic group of strains defined as closely related to Ab33405 is indicated with a green box.

Figure S3. Phylogeny *Acinetobacter baumannii*. Maximum-likelihood phylogenetic tree of closely related strains to Ab33405. Among these strains we found almost all strains annotated as *A. baumannii* species. 1038 strains were used for this phylogenetic analysis. 164 orthologous genes were independently aligned and trimmed. SNPs were extracted from the concatenated sequences and used for phylogenetic analysis. Node support was estimated using the RELL bootstrapping technique in RAXML. The strains selected for genome comparison visualization (Figure 1) belongs to the monophyletic group indicated with a blue box. The red line defines the *A. baumannii* monophyletic cluster.

Figure S4. Comparison of the genetic structure of Ab33405 and *A. calcoaceticus* NCTC 7364 of the locus responsible for the synthesis of the siderophore baumannoferrin. The graphic representation was made using the EasyFig 2.2.2 software.

Figure S5. Schematic representation of the genetic context of IS26 and ISAba31 in Ab33405 genome. The (*) represented a putative lipoprotein sequence and (#) represented a phage hypothetical protein sequence.

Figure S6. Schematic representation of the localization and structure of different phage sequences found in Ab33405 genome. The graphic representation was made using the EasyFig 2.2.2 software and Circos 0.69 software.

Figure S7. Flow chart of the general procedures used in this study.

Figure S8. Bioinformatic pipeline.

Table S1. Genomes used to perform the genomic comparison of Ab33405.

Table S2. Reference number and name protein of the mapped genes showed in Fig. 1.

Table S3. Strain sequences analyzed in this study.

Table S4. List of conserved genes used for sampling genomes based on genomic diversity.

Figure S1.

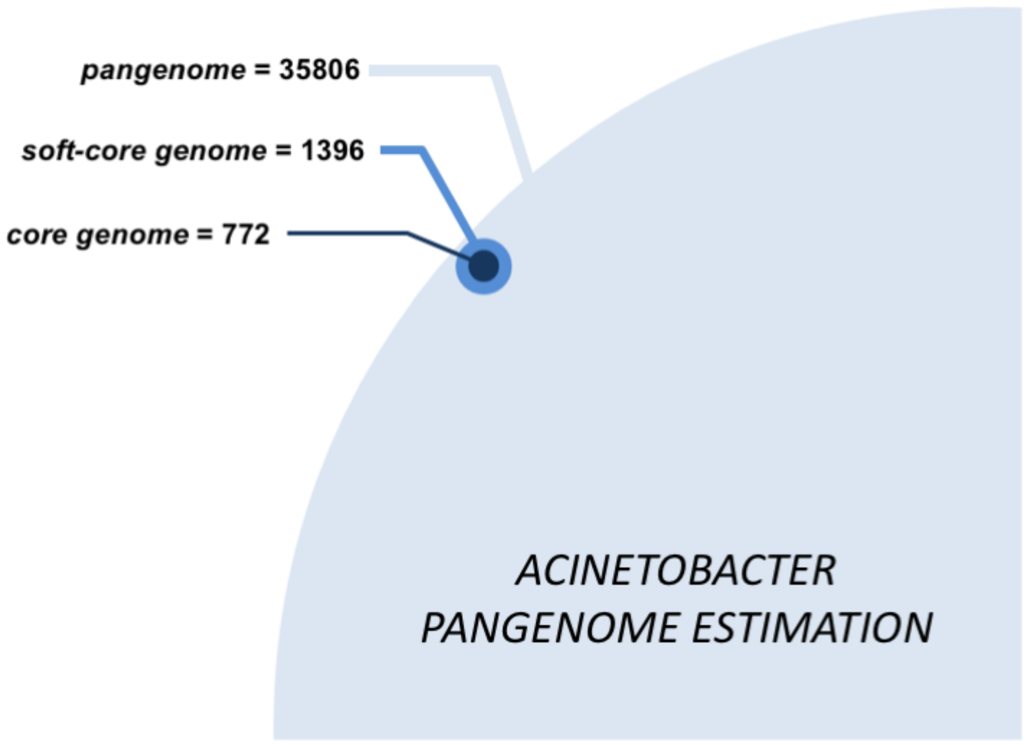
pangenome = 35806
soft-core genome = 1396
core genome = 772
ACINETOBACTER
PANGENOME ESTIMATION

Figure S2

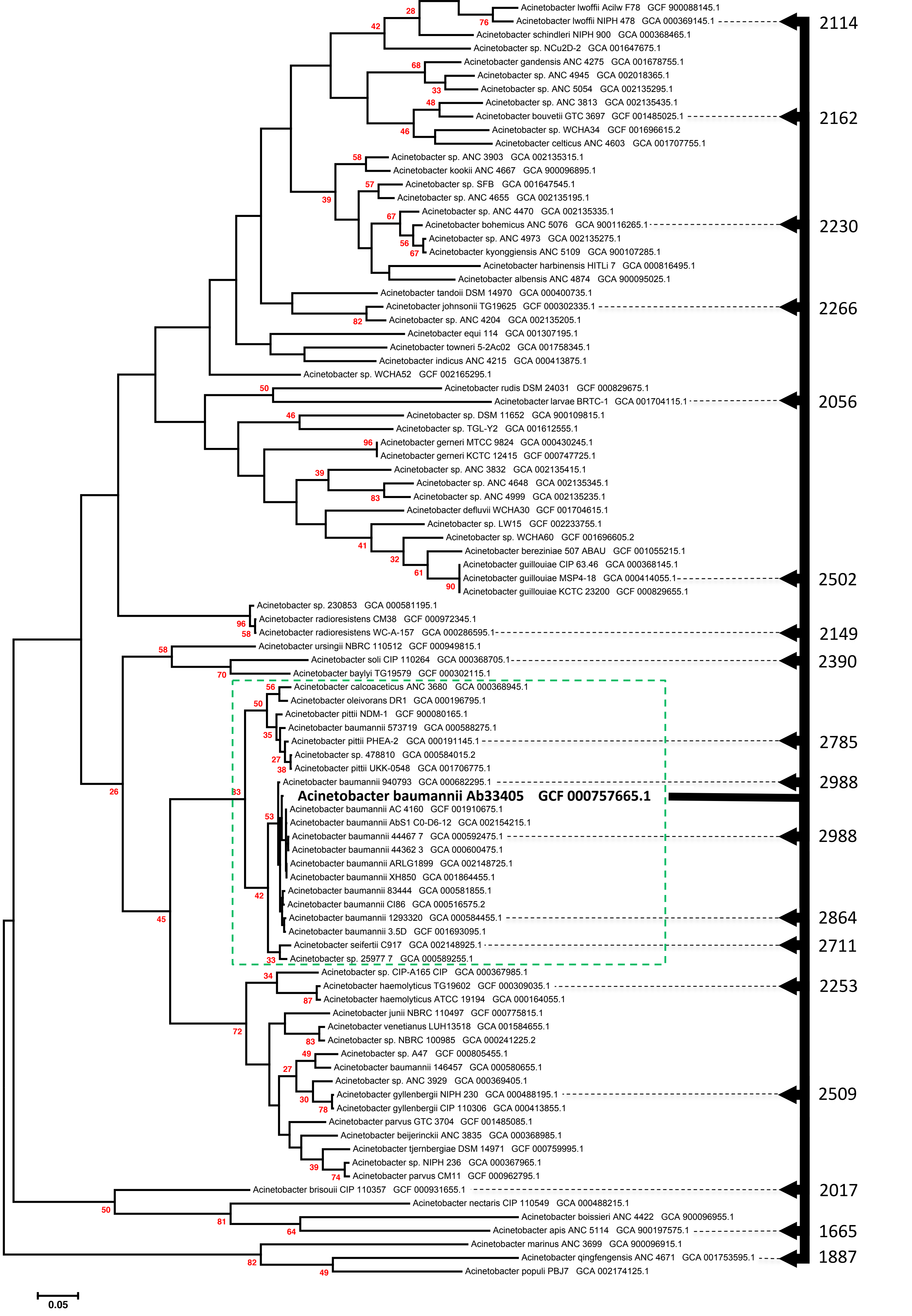

Number of conserved homologous gene families
Acinetobacter sp. NIPH 2171 GCA 000369625.1
Acinetobacter baumannii 348935 GCA 000580795.1
Acinetobacter lwoffii Acilw F78 GCF 900088145.1
Acinetobacter lwoffii NIPH 478 GCA 000369145.1
2114
Acinetobacter schindleri NIPH 900 GCA 000368465.1
Acinetobacter sp. NCu2D-2 GCA 001647675.1
Acinetobacter gandensis ANC 4275 GCA 001678755.1
Acinetobacter sp. ANC 4945 GCA 002018365.1
Acinetobacter sp. ANC 5054 GCA 002135295.1
Acinetobacter sp. ANC 3813 GCA 002135435.1
Acinetobacter bouvetii GTC 3697 GCF 001485025.1
2162
Acinetobacter sp. WCHA34 GCF 001696615.2
Acinetobacter celticus ANC 4603 GCA 001707755.1
Acinetobacter sp. ANC 3903 GCA 002135315.1
Acinetobacter kookii ANC 4667 GCA 900096895.1
Acinetobacter sp. SFB GCA 001647545.1
Acinetobacter sp. ANC 4655 GCA 002135195.1
Acinetobacter sp. ANC 4470 GCA 002135335.1
Acinetobacter bohemicus ANC 5076 GCA 900116265.1
2230
Acinetobacter sp. ANC 4973 GCA 002135275.1
Acinetobacter kyonggiensis ANC 5109 GCA 900107285.1
Acinetobacter harbinensis HITLi 7 GCA 000816495.1
Acinetobacter albensis ANC 4874 GCA 900095025.1
Acinetobacter tandoii DSM 14970 GCA 000400735.1
Acinetobacter johnsonii TG19625 GCF 000302335.1
2266
Acinetobacter sp. ANC 4204 GCA 002135205.1
Acinetobacter equi 114 GCA 001307195.1
Acinetobacter towneri 5-2Ac02 GCA 001758345.1
Acinetobacter indicus ANC 4215 GCA 000413875.1
Acinetobacter sp. WCHA52 GCF 002165295.1
Acinetobacter rudis DSM 24031 GCF 000829675.1
Acinetobacter larvae BRTC-1 GCA 001704115.1
2056
Acinetobacter sp. DSM 11652 GCA 900109815.1
Acinetobacter sp. TGL-Y2 GCA 001612555.1
Acinetobacter gerneri MTCC 9824 GCA 000430245.1
Acinetobacter gerneri KCTC 12415 GCF 000747725.1
Acinetobacter sp. ANC 3832 GCA 002135415.1
Acinetobacter sp. ANC 4648 GCA 002135345.1
Acinetobacter sp. ANC 4999 GCA 002135235.1
Acinetobacter defluvii WCHA30 GCF 001704615.1
Acinetobacter sp. LW15 GCF 002233755.1
Acinetobacter sp. WCHA60 GCF 001696605.2
Acinetobacter bereziniae 507 ABAU GCF 001055215.1
Acinetobacter guillouiae CIP 63.46 GCA 000368145.1
Acinetobacter guillouiae MSP4-18 GCA 000414055.1
2502
Acinetobacter guillouiae KCTC 23200 GCF 000829655.1
Acinetobacter sp. 230853 GCA 000581195.1
Acinetobacter radioresistens CM38 GCF 000972345.1
Acinetobacter radioresistens WC-A-157 GCA 000286595.1
2149
Acinetobacter ursingii NBRC 110512 GCF 000949815.1
Acinetobacter soli CIP 110264 GCA 000368705.1
2390
Acinetobacter baylyi TG19579 GCF 000302115.1
Acinetobacter calcoaceticus ANC 3680 GCA 000368945.1
Acinetobacter oleivorans DR1 GCA 000196795.1
Acinetobacter pittii NDM-1 GCF 900080165.1
Acinetobacter baumannii 573719 GCA 000588275.1
Acinetobacter pittii PHEA-2 GCA 000191145.1
2785
Acinetobacter sp. 478810 GCA 000584015.2
Acinetobacter pittii UKK-0548 GCA 001706775.1
Acinetobacter baumannii 940793 GCA 000682295.1
2988
Acinetobacter baumannii Ab33405 GCF 000757665.1
Acinetobacter baumannii AC 4160 GCF 001910675.1
Acinetobacter baumannii AbS1 C0-D6-12 GCA 002154215.1
Acinetobacter baumannii 44467 7 GCA 000592475.1
2988
Acinetobacter baumannii 44362 3 GCA 000600475.1
Acinetobacter baumannii ARLG1899 GCA 002148725.1
Acinetobacter baumannii XH850 GCA 001864455.1
Acinetobacter baumannii 83444 GCA 000581855.1
Acinetobacter baumannii CI86 GCA 000516575.2
Acinetobacter baumannii 1293320 GCA 000584455.1
2864
Acinetobacter baumannii 3.5D GCF 001693095.1
Acinetobacter seifertii C917 GCA 002148925.1
2711
Acinetobacter sp. 25977 7 GCA 000589255.1
Acinetobacter sp. CIP-A165 CIP GCA 000367985.1
Acinetobacter haemolyticus TG19602 GCF 000309035.1
2253
Acinetobacter haemolyticus ATCC 19194 GCA 000164055.1
Acinetobacter junii NBRC 110497 GCF 000775815.1
Acinetobacter venetianus LUH13518 GCA 001584655.1
Acinetobacter sp. NBRC 100985 GCA 000241225.2
Acinetobacter sp. A47 GCF 000805455.1
Acinetobacter baumannii 146457 GCA 000580655.1
Acinetobacter sp. ANC 3929 GCA 000369405.1
Acinetobacter gyllenbergii NIPH 230 GCA 000488195.1
2509
Acinetobacter gyllenbergii CIP 110306 GCA 000413855.1
Acinetobacter parvus GTC 3704 GCF 001485085.1
Acinetobacter beijerinckii ANC 3835 GCA 000368985.1
Acinetobacter tjernbergiae DSM 14971 GCF 000759995.1
Acinetobacter sp. NIPH 236 GCA 000367965.1
Acinetobacter parvus CM11 GCF 000962795.1
Acinetobacter brisouii CIP 110357 GCF 000931655.1
2017
Acinetobacter nectaris CIP 110549 GCA 000488215.1
Acinetobacter boissieri ANC 4422 GCA 900096955.1
Acinetobacter apis ANC 5114 GCA 900197575.1
1665
Acinetobacter marinus ANC 3699 GCA 900096915.1
Acinetobacter qingfengensis ANC 4671 GCA 001753595.1
1887
Acinetobacter populi PBJ7 GCA 002174125.1
0.05

Figure S3

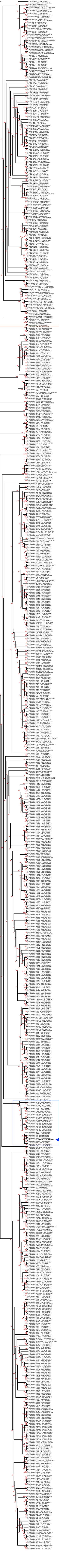

Figure S4

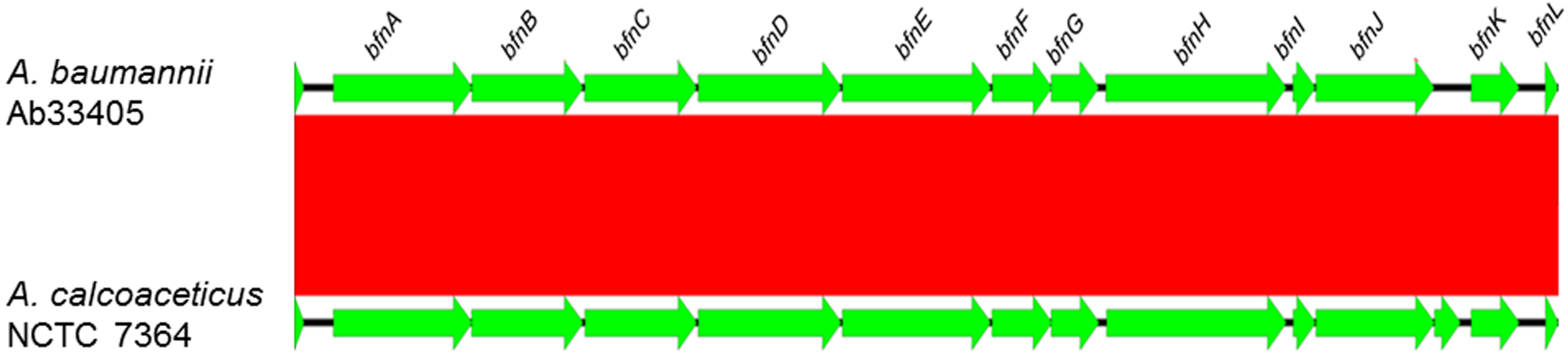

bfnA
bfnB
bfnC
bfnD
bfnE
bfnF
bfnG
bfnH
bfnI
bfnJ
bfnK
bfnL
A. baumannii Ab33405
A. calcoaceticus NCTC 7364

Figure S5

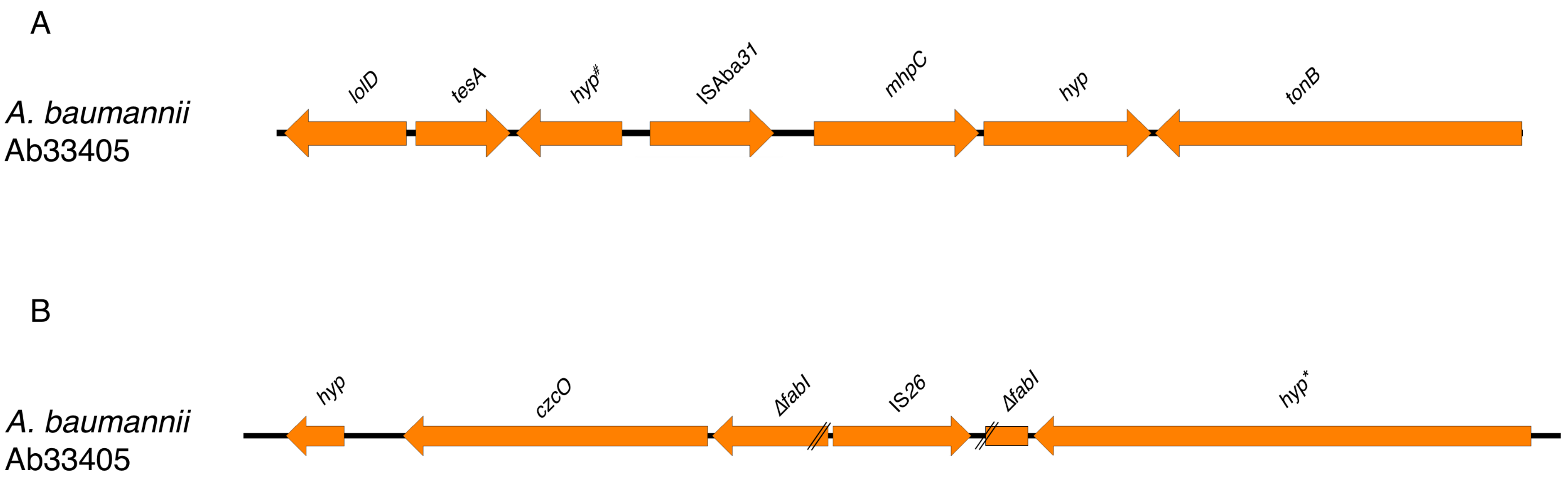
A
A. baumannii
Ab33405
lolD
tesA
hyp#
ISAba31
mhpC
hyp
tonB
B
A. baumannii
Ab33405
hyp
czcO
ΔfabI
IS26
ΔfabI
hyp*

Figure S6

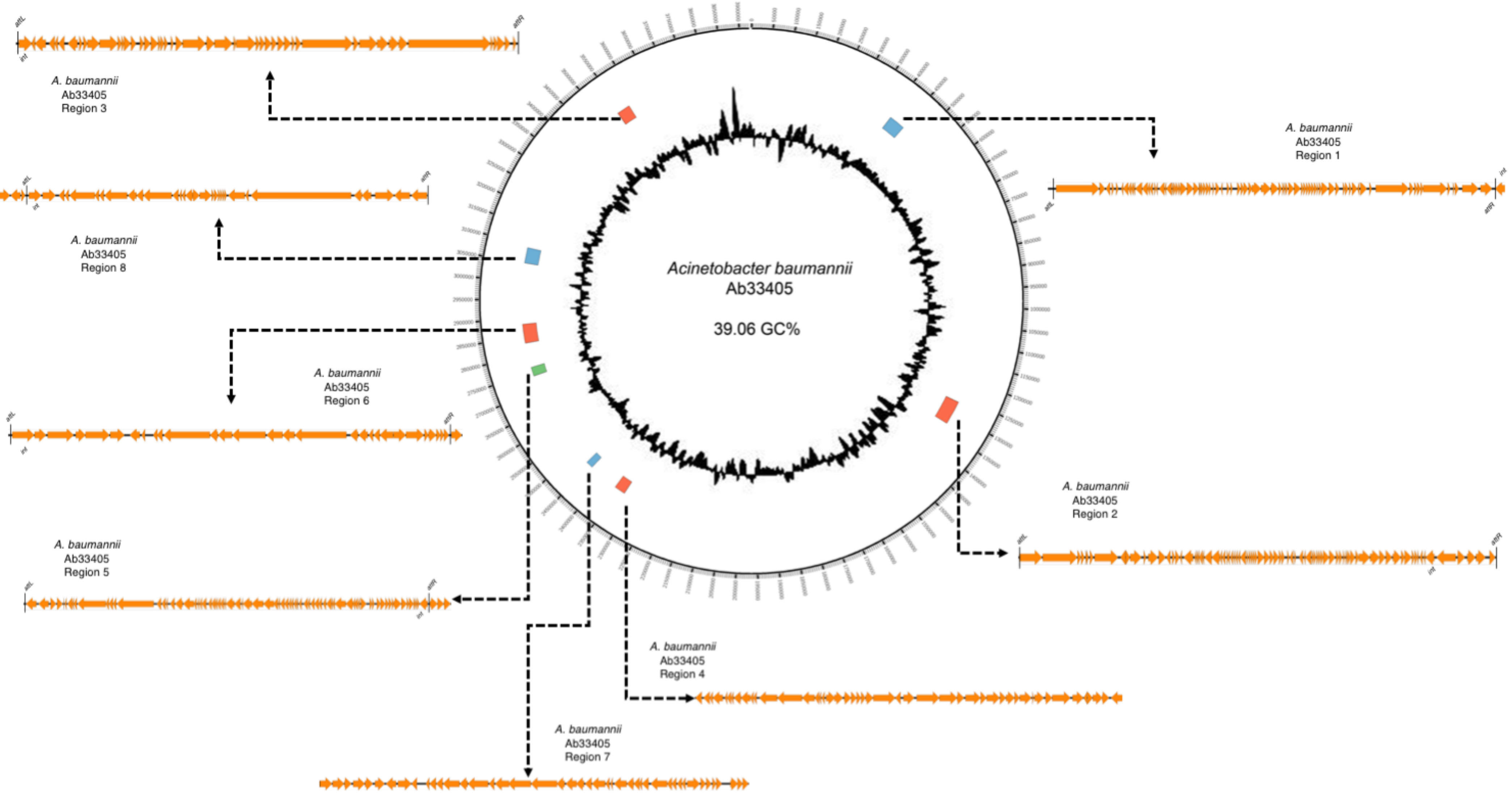

Acinetobacter baumannii
Ab33405
39.06 GC%
A. baumannii
Ab33405
Region 1
A. baumannii
Ab33405
Region 2
A. baumannii
Ab33405
Region 3
A. baumannii
Ab33405
Region 4
A. baumannii
Ab33405
Region 5
A. baumannii
Ab33405
Region 6
A. baumannii
Ab33405
Region 7
A. baumannii
Ab33405
Region 8

Figure S7

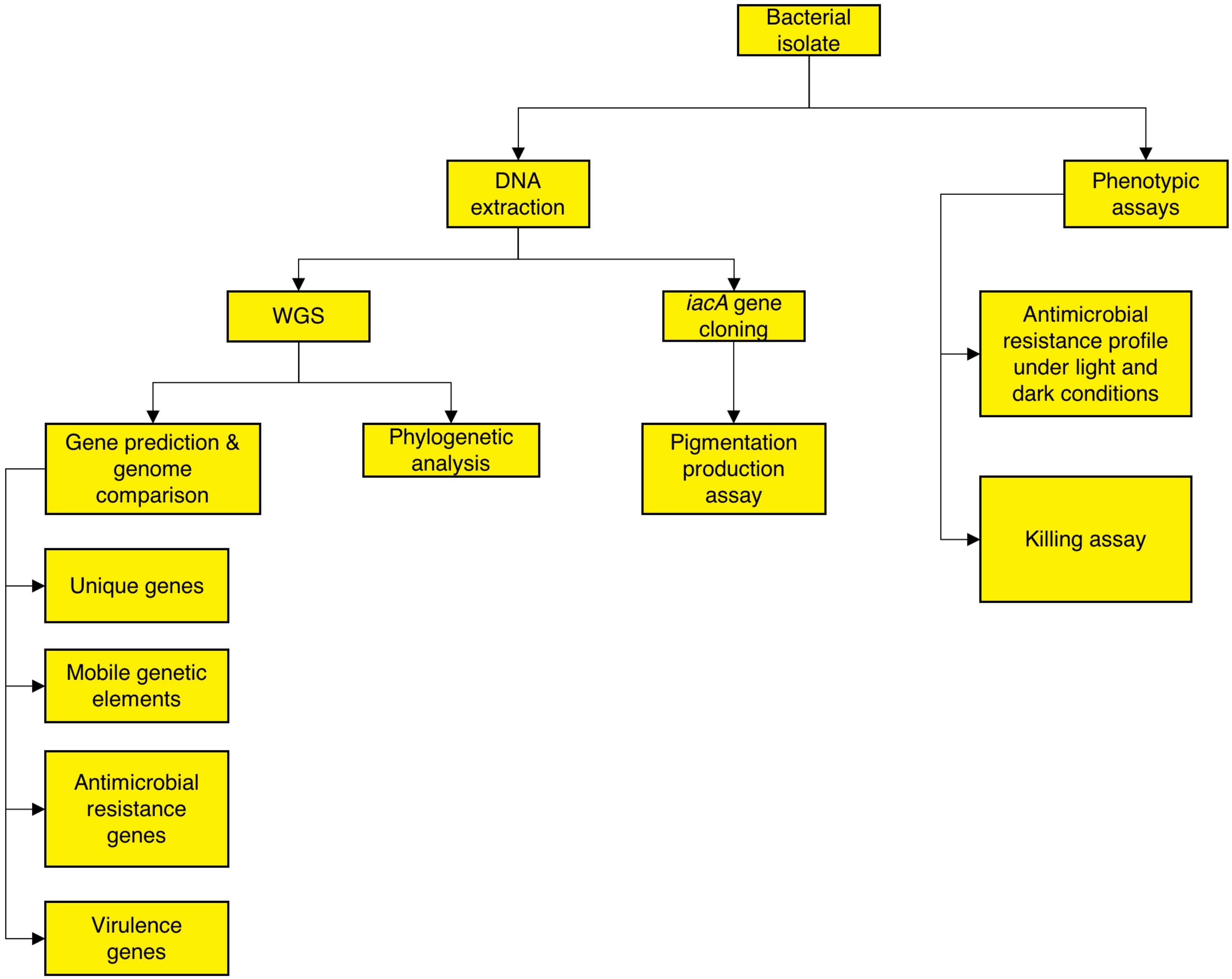

Bacterial isolate
DNA extraction
Phenotypic assays
WGS
iacA gene cloning
Antimicrobial resistance profile under light and dark conditions
Gene prediction & genome comparison
Phylogenetic analysis
Pigmentation production assay
Killing assay
Unique genes
Mobile genetic elements
Antimicrobial resistance genes
Virulence genes

Figure S8

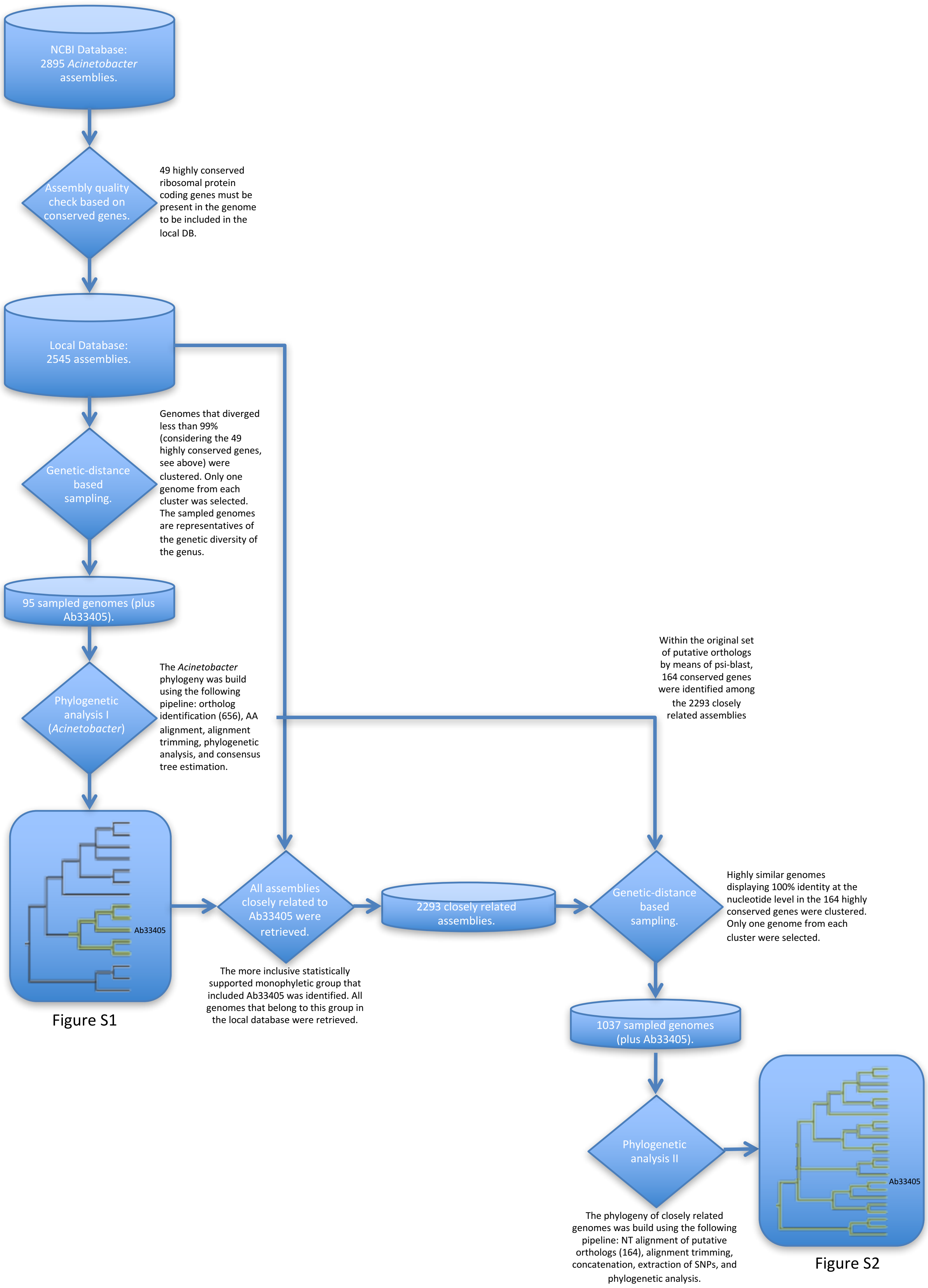
NCBI Database:
2895 *Acinetobacter* assemblies.
Assembly quality check based on conserved genes.
49 highly conserved ribosomal protein coding genes must be present in the genome to be included in the local DB.
Local Database:
2545 assemblies.
Genetic-distance based sampling.
Genomes that diverged less than 99% (considering the 49 highly conserved genes, see above) were clustered. Only one genome from each cluster was selected. The sampled genomes are representatives of the genetic diversity of the genus.
95 sampled genomes (plus Ab33405).
Phylogenetic analysis I (*Acinetobacter*)
The *Acinetobacter* phylogeny was build using the following pipeline: ortholog identification (656), AA alignment, alignment trimming, phylogenetic analysis, and consensus tree estimation.
Ab33405
Figure S1
All assemblies closely related to Ab33405 were retrieved.
The more inclusive statistically supported monophyletic group that included Ab33405 was identified. All genomes that belong to this group in the local database were retrieved.
2293 closely related assemblies.
Within the original set of putative orthologs by means of psi-blast, 164 conserved genes were identified among the 2293 closely related assemblies
Genetic-distance based sampling.
Highly similar genomes displaying 100% identity at the nucleotide level in the 164 highly conserved genes were clustered. Only one genome from each cluster were selected.
1037 sampled genomes (plus Ab33405).
Phylogenetic analysis II
The phylogeny of closely related genomes was build using the following pipeline: NT alignment of putative orthologs (164), alignment trimming, concatenation, extraction of SNPs, and phylogenetic analysis.
Ab33405
Figure S2

Table S1

Genome	sizes (Mb[a])	GC%	N° CDSs[b]	[c]ST (PI /OU)	Accession Number
**AYE**	4.04873	39.34	3725	ST1 / ST231	CU459141
**1656-2**	4.02311	39.08	3733	ST2 / ST423	CP001921
**AB307-0294**	3.76098	39	3363	ST1 / ST231	CP001172
**AB030**	4.33579	39	4155	ST79 / ST758	CP009257
**AB031**	3.80332	39.20	3472	ST638 / ST1000	CP009256
**AB0057**	4.05924	39.19	3669	ST1 / ST207	CP001182
**AbH12O-A2**	3.87577	39.10	3673	ST79 / ST924	CP009534
**ACICU**	3.99676	38.90	3802	ST2 / ST427	CP000863
**MDR-TJ**	4.15341	39.07	4011	ST2 / ST369	CP003500
**MDR-ZJ06**	4.01143	39.04	3688	ST2 / ST643	CP001937
**TCDC-AB0715**	4.13079	38.9	3844	ST2 / ST218	CP002522
**TYTH-1**	3.95737	39	3628	ST2 / ST455	CP003856
**LAC-4**	3.96844	38.99	3633	ST10 / ST447	CP007712
**IOMTU433**	4.19032	39.12	3868	ST622 / ST919	AP014649
**D1279779**	3.7117	38.99	3524	ST267 / ST942	CP003967
**ATCC17978**	4.0067	38.94	3673	ST437 / ST112	CP000521
**BJAB0715**	4.05389	38.92	3756	ST23 / ST642	CP003847

[a]Mb, Megabase.
[b]CDS, coding sequences.
[c]ST: Type Sequence; PI MLST scheme of the Pasteur Institute; OU: MLST scheme of the University of Oxford.
